# Carbonic anhydrase XII as biomarker and therapeutic target in ovarian carcinomas

**DOI:** 10.1371/journal.pone.0271630

**Published:** 2022-07-28

**Authors:** Lisa Hiepp, Doris Mayr, Kathrin Gärtner, Elisa Schmoeckel, Frederick Klauschen, Alexander Burges, Sven Mahner, Reinhard Zeidler, Bastian Czogalla

**Affiliations:** 1 Institute of Pathology, Ludwig-Maximilians-University Munich, Munich, Germany; 2 Research Group Therapeutic Antibodies, Helmholtz Center Munich–German Research Center for Environmental Health, Munich, Germany; 3 Department of Obstetrics and Gynecology, University Hospital, Ludwig-Maximilians-University Munich, Munich, Germany; 4 Department of Otorhinolaryngology, University Hospital, Ludwig-Maximilians-University Munich, Munich, Germany; Cleveland Clinic Lerner Research Institute, UNITED STATES

## Abstract

Targeting the tumor-associated carbonic anhydrase XII (CA XII) is considered a promising strategy to improve cancer treatment. As such progress is highly demanded for ovarian carcinomas, the present study aimed to provide deeper information about their CA XII expression profile. A large collection of tissue specimens was stained immunohistochemically with a specific anti-CA XII antibody to evaluate the expression in neoplastic and non-neoplastic epithelial ovarian cells. In addition, flow cytometry was used to measure CA XII expression on tumor cells from malignant ascites fluid. Binding of the antibody revealed a significant CA XII expression in most ovarian carcinoma tissue samples and ascites-derived ovarian carcinoma cells. Moreover, CA XII was expressed at higher levels in ovarian carcinomas as compared to borderline ovarian tumors and non-neoplastic ovarian epithelia. Within the carcinoma tissues, high expression of CA XII was associated with higher tumor grading and a trend towards shorter overall survival. Our results indicate that CA XII plays a crucial role for the malignancy of ovarian carcinoma cells and emphasize the potential of CA XII as a diagnostic marker and therapeutic target in the management of ovarian carcinomas.

## Introduction

Ovarian cancer is the most lethal gynecological tumor type that annually accounts for more than 200,000 deaths worldwide, numbers increasing [1, 2]. The poor prognosis is attributable to the lack of reliable screening methods, the absence of specific symptoms until advanced stage disease, and limited therapeutic options in recurrent and progressive ovarian cancer. Consequently, there is a high need for new diagnostic and prognostic markers, and therapeutic targets [3]. Ovarian carcinomas (OCs) constitute about 90% of the ovarian malignancies and are classified into histological subtypes of considerable biological heterogeneity with high-grade serous carcinomas (HGSCs) representing the most common type [4, 5].

In search of new strategies for tumor treatment, targeting cell metabolism is subject to intensive research [6]. Carbonic anhydrases (CAs) are zinc metalloenzymes that catalyze the reversible hydration of CO_2_ to bicarbonate and protons and are therefore involved in vital physiological processes. CA XII is a membrane isozyme that is upregulated in several tumor types [7–9]. Typically, malignant cells cover a part of their high energetic demand via glycolysis, resulting in the accumulation of acidic side products like lactate. CA XII is crucial for the regulation of the intracellular pH and thus for the maintenance of cell function and survival. Additionally, the enzyme contributes to the acidification of the tumor microenvironment, which in turn promotes tumor invasion and migration [10–12]. Consequently, CA XII is considered an attractive druggable target for specific therapies with either blocking antibodies [13–18] or small molecules [19–23].

In the present study, we analyzed the expression of CA XII in neoplastic and non-neoplastic ovarian cells on tissue specimens and in malignant ascites fluid using the antibody 6A10 [13]. By investigating differences in expression and correlating the findings with clinicopathological parameters, we aimed to contribute to new approaches for OC treatment.

## Materials and methods

### Patients and specimens

Tissue samples from 487 patients, who underwent surgery at the Department of Obstetrics and Gynecology, University Hospital, Ludwig-Maximilians-University (LMU) Munich between 1990 and 2019, were included in this study. Specimens were diagnosed as OC (*n* = 456) or borderline ovarian tumor (BOT, *n* = 20) by specialized pathologists at the Institute of Pathology, LMU Munich. Non-neoplastic ovaries served as a control group (CG, *n* = 20). The predominant cohort consisted of primarily diagnosed OCs with specimens available on tissue microarrays (TMAs), together with corresponding histopathological and clinical data. Carcinomas were graded and typed according to the WHO classification of 2014. No patient had received neoadjuvant chemotherapy. Analyzed patients’ data are summarized in Table 1. One paraffin tissue block per patient existed for the comparison cohorts (BOT, CG), and data were transferred from pathological reports on diagnostic findings. Additionally, 22 samples of ascites fluid with tumor cells were obtained from OC patients treated at the Department of Obstetrics and Gynecology, University Hospital, LMU Munich, as well as two samples of patients with BOT between 2015 and 2019.

**Table 1:** Patient characteristics of the analyzed OC tissue samples.

Parameters	Number	Percentage (%)
Histology		
Serous, low-grade	40	10.2
Serous, high-grade	267	68.1
Endometrioid	37	9.4
Mucinous	17	4.3
Seromucinous	4	1.0
Clear cell	15	3.8
Undifferentiated	8	2.0
Missing	4	1.0
Grading ^a^		
G1	57	14.5
G2	28	7.1
G3	301	76.8
GX/missing	6	1.5
Primary tumor expansion		
T1	66	16.8
T2	35	8.9
T3	282	71.9
TX/missing	9	2.3
Nodal status		
N0	109	27.8
N1	125	31.9
NX/missing	158	40.3
Distant metastasis		
M0	13	3.3
M1	41	10.5
MX/missing	338	86.2
FIGO		
I	56	14.3
II	25	6.4
III	266	67.9
IV	41	10.5
Missing	4	1.0
Median age, range (years)	62, 23–93	

^a^ not considering histological type

### Immunohistochemistry

3-μm-thick sections were cut from the paraffin blocks and mounted on Q Path adhesive slides (VWR International). A modified staining protocol was established due to the first usage of the antibody 6A10 [13] on paraffin-embedded tissue sections. The staining procedure was identical for all specimens and performed as follows:

- deparaffinization in xylol and rehydration in a descending ethanol series;
- heat pretreatment in the microwave with Target Retrieval Solution Citrate pH 6 (Agilent Technologies, S2369);
- quenching endogenous peroxidase with 7.5% H_2_O_2_ for 10 min;
- prevention of non-specific binding with Protein Block Serum-Free Ready-to-use (Agilent Technologies, X0909) for 10 min;
- incubation with anti-CA XII primary antibodies (1:5 dilution, monoclonal rat IgG2a, clone 6A10 [13], antibody concentrate, Core Facility ’Monoclonal Antibodies’ at the Helmholtz-Center Munich) for 60 min at room temperature;
- application of detection system SuperVision 2 Single Species HRP-Polymer Rat (DCS, PD000POL-R) by incubating with SuperVision 2 Enhancer for 20 min and then with SuperVision 2 Polymer-HRP for 30 min;
- staining with substrate and chromogen 3,3’-diaminobenzidine (DAB+, Agilent Technologies, K3468) for 10 min, followed by counterstaining with Hematoxylin Gill’s Formula (Vector Laboratories, H-3401).

System and isotype controls were included to assess the specificity of the immunoreactions.

### Staining evaluation

Carcinoma cells of 392 cases (85.96%) were still available for examination after the staining process. In the comparison cohorts, 19 cases (95%) remained evaluable for BOT and CG, respectively. Staining was rated in the entirety of epithelial cells conformable with the decisive histological diagnosis for the cohort classification. Inclusion cysts found in some ovaries were therefore treated as surface epithelium. Embryonic remnants were not considered. To provide comparableness, evaluation of all immunohistochemically stained tissues was carried out under identical conditions on a LEICA microscope (DM 2500). The assessment of CA XII staining was based on the immunoreactive score (IRS, Remmele score [24]), which is a semi-quantitative method combining the percentage of positive-stained cells (PP) and the predominant staining intensity (SI). By multiplying the assigned value of PP (0: no staining, 1: <10%, 2: 10–50%, 3: 51–80%, 4: >80% of the cells) and SI (0: no, 1: weak, 2: moderate, 3: strong staining (S1 Fig)), an IRS between 0 and 12 was obtained. CA XII staining with an IRS of >2 was regarded as positive.

### Flow cytometry

CA XII expression on the tumor cells isolated from ascites fluid was measured by flow cytometry using Alexa647-labeled 6A10 [13] (Core Facility ’Monoclonal Antibodies’ at the Helmholtz-Center Munich) following standard staining protocols. Cells were analyzed with a Becton-Dickinson FACSCanto. Data were analyzed with the FlowJo software.

### Statistics

Statistical analysis and visualization of data were performed employing IBM SPSS Statistics version 26. Pictures of tissue samples were taken with a LEICA DMD 108 photomicroscope. All samples could be regarded as unpaired, and as the CA XII expression showed no normal distribution, non-parametric tests were applied. Due to the limited number of possible values, the IRS was rather considered as a nominal variable. Therefore, Pearson’s chi-squared test or Fisher’s exact test was used to compare CA XII expression between the cohorts and to identify correlations with pathological and clinical variables within the carcinoma subgroup. Effect sizes were calculated using Cramer’s V (range 0–1; with 0.1 = weak, 0.3 = moderate and 0.5 = strong effect size). Patients’ follow-up data were available as overall survival (OS), and Kaplan–Meier curves and log-rank testing were performed to compare survival times according to different levels of CA XII expression. All tests were two-tailed and statistical significance was assumed for *p* < 0.05. Bonferroni correction was applied to adjust multiple testing where necessary.

### Ethical approval

Specimens included in this study were obtained from leftover material remaining after the completed histopathological examination. Patients’ data were fully anonymized, and the authors were blinded for clinical information during the analysis and could not identify individual participants during or after data collection. Due to these described circumstances the Ethics Committee of the LMU Munich declared that no written informed consent of the participants or permission to publish is needed. The study was performed between 2019 and 2021 according to the standards set in the Declaration of Helsinki 1975 and approved by the Ethics Committee of the LMU Munich (approval numbers 089–13, 227–09, 19–972, and 20–025).

## Results

### CA XII expression in OC, BOT and CG

**Fig 1:**
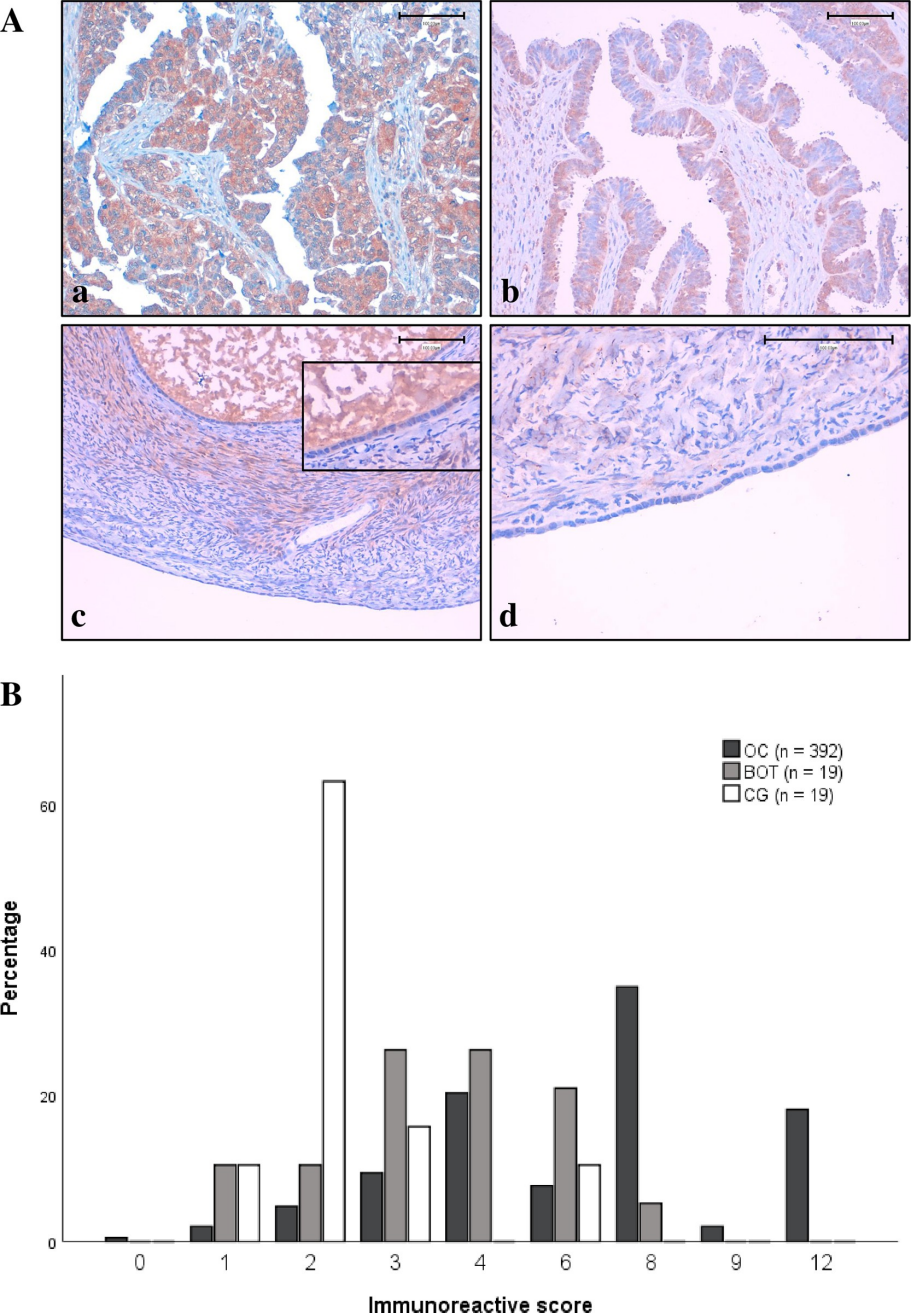
CA XII expression across cohorts. (A) Immunohistochemical detection of CA XII in OC (a), BOT (b), ovarian cortex with inclusion cyst (c), and surface epithelium (c, d). ×200 magnification was used for a, b, c, and ×400 magnification for d (scale bars: 100 μm). (B) Distribution of IRS values. The y-axis indicates the percentage within each cohort.

Positive staining (defined as IRS >2) was observed in 92.6% of evaluable OC cases (363 out of 392). In most positive cases, CA XII expression was present in more than eighty percent (PP4) of the available tumor tissue. Such staining of the OC tissue is exemplified in Fig 1A panel ’a’. The distribution of IRS values in the OC cohort is visualized with black bars in Fig 1B. The median IRS was 8 and the range of IRS was 0–12 with only two cases staining completely negative. Explicitly heterogeneous staining was observed within the tissue specimens of some patients. Areas of strong staining intensity were present in 69.4% of cases. Even if the plasma membrane was accentuated in several cells, staining was mainly intracellular, and especially when staining was sparse, a nuclear expression became apparent in a few specimens. Staining apart from the epithelial cells was detected in various cases, for example in stromal cells or psammoma bodies.

Expression of CA XII was also observed in the semi-malignant BOTs (Fig 1B, grey bars) and in the non-neoplastic CG (Fig 1B, white bars), where staining was sometimes confined to the basal part of the epithelium, adjacent to the stroma. Fig 1A opposes the OC staining (panel ’a’) to examples of BOT tissue (panel ’b’) and non-neoplastic ovarian tissue (panels ’c’ and ’d’). There was no fundamental difference in staining between ovarian surface epithelium and the epithelial cells of inclusion cysts.

Overall, significantly higher IRS values were documented for the OC cohort (Fig 1, Table 2). The percentage of positive-stained cells was significantly increased in OC as compared to BOT (*p* < 0.001) and even more as compared to CG (*p* < 0.001). Staining in BOT and CG mainly differed in intensity (*p* = 0.017), resulting in higher IRS values for the BOT tissue (*p* = 0.004). Moreover, tissues of low-grade serous carcinoma (LGSC), serous BOT and CG were compared to each other. Staining parameters showed significant differences between the same pairs of comparison as described for the complete cohorts.

**Table 2:** Summarized *p*-values (effect sizes in brackets) of compared CA XII expression between the cohorts.

	IRS	PP	SI
OC vs. BOT	< 0.001* (0.246)	< 0.001* (0.319)	0.102 (0.127)
OC vs. CG	< 0.001* (0.383)	< 0.001* (0.380)	< 0.001* (0.211)
BOT vs. CG	0.004* (0.615)	0.306 (0.306)	0.017 (0.436)

Statistical significance (indicated by asterisks) was assumed for *p* < 0.0167 due to Bonferroni correction. IRS = immunoreactive score, PP = percentage of positive-stained cells, SI = staining intensity.

### Correlation of CA XII expression to clinicopathological parameters

Regarding the entire cohort of OCs, particularly high IRS values were distributed differently between grades of tumor cell differentiation. Statistical significance essentially resulted from the different incidences of strong SI (*p* = 0.005), including the absence of IRS 12 in low-grade carcinomas. The impact of the histological type on CA XII expression could not be reasonably investigated due to the small case number of single OC subtypes and differing tumor grade. Other available parameters were tested on HGSCs. No correlation with CA XII expression could be identified regarding T, N, M, FIGO, or age. Table 3 shows the conducted correlations with a grouped IRS, considering the relevance of high IRS values mentioned above.

**Table 3:** Correlation analysis within the OC cohort regarding grouped immunoreactive score (0–2, 3–8, 9–12).

Variables	*n*	*p* ^a^	Effect sizes
Grading (all histological subtypes)	386	0.007*	0.187
Grading serous (LGSC vs. HGSC)	307	0.007*	0.175
Primary tumor expansion	263	0.441	0.114
Nodal status	267	0.706	0.091
Distant metastasis	267	0.126	0.155
FIGO	265	0.077	0.197
Age (< median vs. > median)	266	0.807	0.046

^a^ Statistical significance is indicated by asterisks.

### Correlation of CA XII expression to overall survival

**Fig 2:**
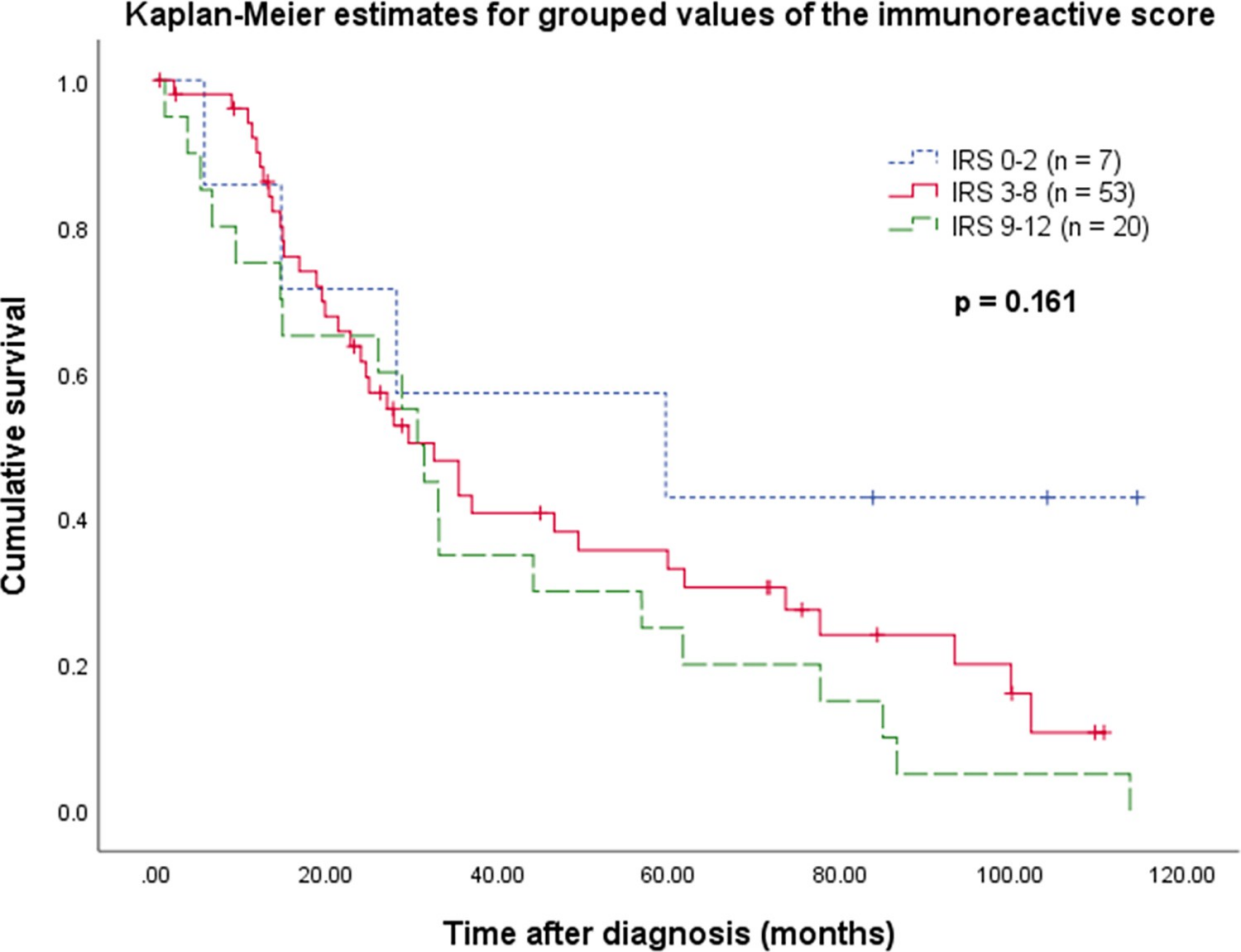
Overall survival concerning CA XII expression within a stratified subgroup of OC patients. Censoring events have been marked in the graph.

To evaluate a potential correlation between the CA XII expression and patient survival, the OC cohort was stratified by grading, histological type, tumor stage, and age (enclosing the group with the most cases for every parameter), resulting in a subgroup of 80 cases with the following properties: HGSC, FIGO III, 60–69 years old. 61 (76.2%) of these patients died during the observation time and the median OS was 31.4 months. The seven cases with negative IRS (IRS ≤2) tended to have a better OS than the 73 cases with IRS >2, which became apparent approximately after 20 months of follow-up (*p* = 0.123). Moreover, divergence of survival curves was observed at a cutoff IRS >8, with high IRS values showing shorter survival (*p* = 0.144). This was due to the presence or absence of predominantly strong SI. Fig 2 presents the Kaplan-Meier estimates of grouped IRS values (0–2, 3–8, 9–12) pursuant to the described observations (*p* = 0.161). However, the survival curves of tested expression levels did not always show a clear gradation and findings were not statistically significant.

### CA XII expression on tumor cells isolated from ascites fluid

**Fig 3:**
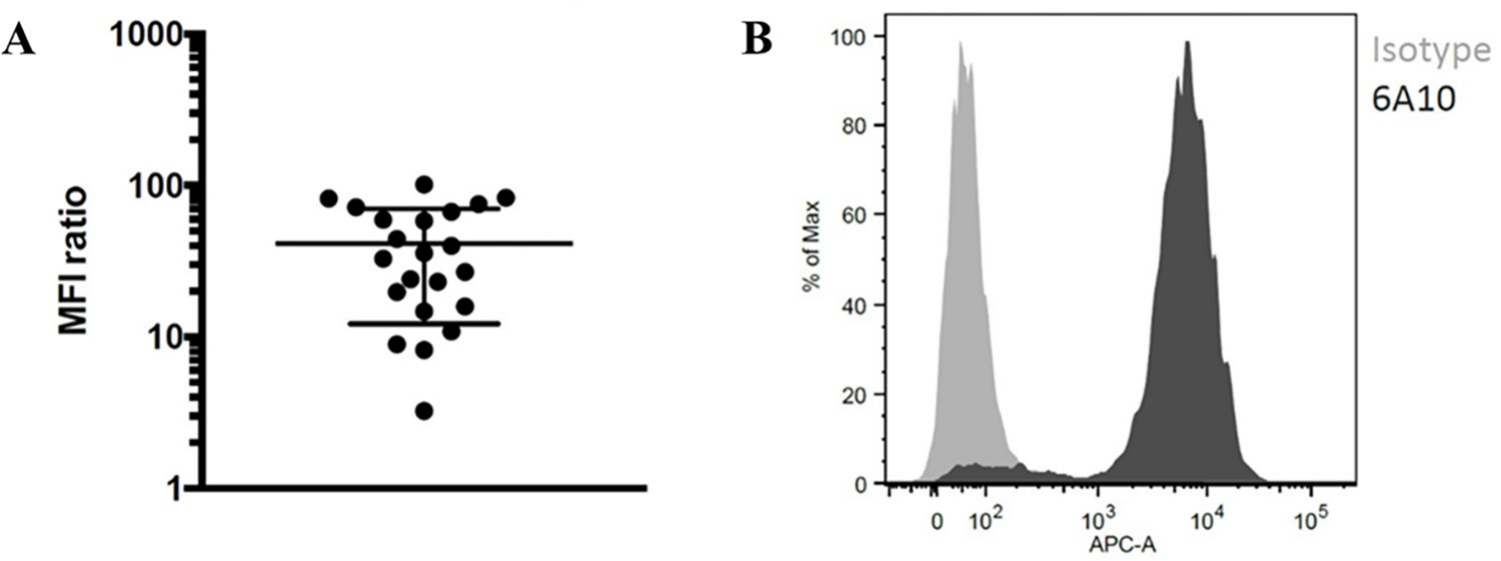
**Detection of CA XII by flow cytometry.** (A) Quantity of CA XII expression on vital, adherent OC cells in ascites samples. (B) Representative histogram of fluorescence measurement.

Significant CA XII expression was also detected in all 22 samples of ascites fluid derived from OC patients, which is demonstrated by Fig 3A. The mean fluorescence identity (MFI) ratio was calculated by dividing the MFI for 6A10 through the MFI for an isotype control antibody (Fig 3B). In addition, two BOT cases were analyzed. In sharp contrast to invasive carcinoma, CA XII expression was virtually absent in both samples (not shown).

## Discussion

OC is one of the deadliest types of cancer. This is also due to the fact that the disease is often only detected at late stages and that biomarkers of risk or early detection are lacking [3]. CA XII is a hypoxia-induced ectoenzyme involved in intracellular pH regulation and overexpressed in many neoplastic tissues, including gynecological tumors [8, 25–27]. Despite this fact, only a few authors have systematically investigated CA XII in OCs and, consequently, only limited data are available so far [8, 26].

In the present study, we used the antibody 6A10 [13] for the staining of a large collection of paraffin-embedded OC tissues. The results revealed that a significant expression of CA XII is detectable in the vast majority of cases. Moreover, CA XII was strongly expressed in all (22 out of 22) analyzed cases of malignant ascites. Variable, albeit generally lower, CA XII expression levels were observed in BOT tissue sections and non-neoplastic epithelial cells of ovarian tissue. There was a significant correlation between the CA XII expression levels and tissue malignancy. Even within the carcinoma group, high expression was associated with poorer tumor cell differentiation. Additionally, high expression at the time of diagnosis showed a trend towards shorter survival.

Our results demonstrate that CA XII is upregulated in the majority of OCs, indicating that the enzyme might be of relevance for OC biology. While inter- and intratumor expression levels varied, probably due to morphological and molecular tumor heterogeneity [4, 5], areas within OC tissues that stained completely negative were only rarely observed. In addition, the distribution of IRS values revealed that high-grade tumors express higher levels of CA XII. As CA XII contributes to the acidification of the microenvironment and thereby promotes tumor spreading [10–12], one could speculate that the enzyme causally contributes to impaired patient survival.

As staining predominantly was cytoplasmic in immunohistochemistry, no predication concerning the mere influence of membranous CA XII expression could be provided. Interestingly, the amount of intracellular CA XII constituted a parameter of relevance in this study. At first sight, the staining of intracellular compartments may question the specificity of antigen detection. Paraffin-embedded sections pose a challenge for the application of antibodies, and unintended side effects of immunostaining can never be excluded. Nevertheless, the presence of membrane-associated molecules in dissenting subcellular localizations is approved [28, 29], and such evidence has already been provided for transmembrane CAs, especially for CA IX [8, 26, 30–34]. Frequently described cytoplasmic staining has either been attributed to the detection of newly produced enzymes [35, 36] or vesicle-trapped enzymes with different extracellular [37] or cellular destinations. Internalization by endocytosis can be an instrument to remove molecules from the cell surface for regeneration or to regulate their abundance. Moreover, it enables membrane-associated molecules to participate in intracellular processes [30, 38]. In 2013, Buanne et al. [31] studied partners of the CA IX molecule and reported its association with proteins of the nuclear transport machinery. They additionally found increased nuclear and perinuclear staining in hypoxic cells.

The impact of hypoxia on CA XII expression could not be investigated in detail within this study. No obvious relation between areas of low oxygen level and the distribution of CA XII expression was noticed on the immunostained tissues, which is in accordance with observations of previous studies [26, 39] and the perception of hypoxia not being the main regulator of CA XII [40]. Consistent with the findings of our research, induction of CA XII was shown to be linked to the grade of cell differentiation. However, association with lower grade is considered for certain tumor entities [25, 41, 42].

Although CA XII was also found to be expressed in some healthy ovarian epithelia, as already reported by Ivanov et al. [8], the expression levels were significantly lower than in carcinomas. The interim position of the BOT provided interesting information, not only considering the grade of nuclear atypia and mitotic activity but also tumor aggressiveness. Despite the atypical proliferation of the epithelium, BOTs are characterized by the absence of destructive stromal invasion and have by far a better clinical prognosis [43, 44]. In line, BOT revealed a significantly lower CA XII expression than malignant carcinoma tissue.

Early detection of OCs would improve patient survival, but no reliable marker is available for an effective screening regimen [3]. Despite its limited specificity and sensitivity for OC diagnosis, the cancer antigen 125 (CA 125) [45] is used for therapy monitoring and recurrence detection [46]. Considering our results obtained from the analysis of ascites, CA XII could be a reliable marker for the detection of OC cells in the peritoneal fluid. In line, the inhibitory property of the antibody 6A10 [13] could additionally contribute to a reduction or even prevention of tumor spreading [14, 15], most likely as part of an Antibody-Drug-Conjugate (ADC) [15, 47]. Since CA XII was found to be expressed in almost all OCs, these carcinoma cells can in principle be readily accessed for therapeutic purposes. A prerequisite for this is the binding of the relevant enzyme domain on the outside of the plasma membrane of vital tumor cells, which could be shown by flow cytometry.

## Conclusions

The presence of CA XII expression in OCs has already been reported by others [8, 26], but further information was needed for the evaluation of CA XII as diagnostic marker or therapeutic target. This study not only investigated the CA XII expression in more detail on a higher number of neoplastic and non-neoplastic ovarian tissues but also analyzed vital ovarian tumor cells from ascites fluid. For the first time, OC cells were examined with the monoclonal antibody 6A10, a promising tool for cancer treatment [13]. Our results indicate that CA XII plays a role for the aggressive behavior of OCs, which might be the rationale for a potential new strategy in the management of OC patients.

In conclusion, we identify CA XII as an attractive target antigen in OCs, a tumor entity with the exigent necessity of new diagnostic and therapeutic options.

## Supporting information

S1 Fig Immunohistochemical staining examples of CA XII expression in high grade serous ovarian carcinomas. (PDF)

S1 Table Minimal data set. (PDF)
