# Supplementary material for: Carbonic anhydrase XII as biomarker and therapeutic target in ovarian carcinomas
Source: PLoS One. 2022 Jul 28;17(7):e0271630. doi: 10.1371/journal.pone.0271630 (PMC9333239; doi:10.1371/journal.pone.0271630)
Supplement: S1 Fig — (PDF) [file pone.0271630.s001.pdf]

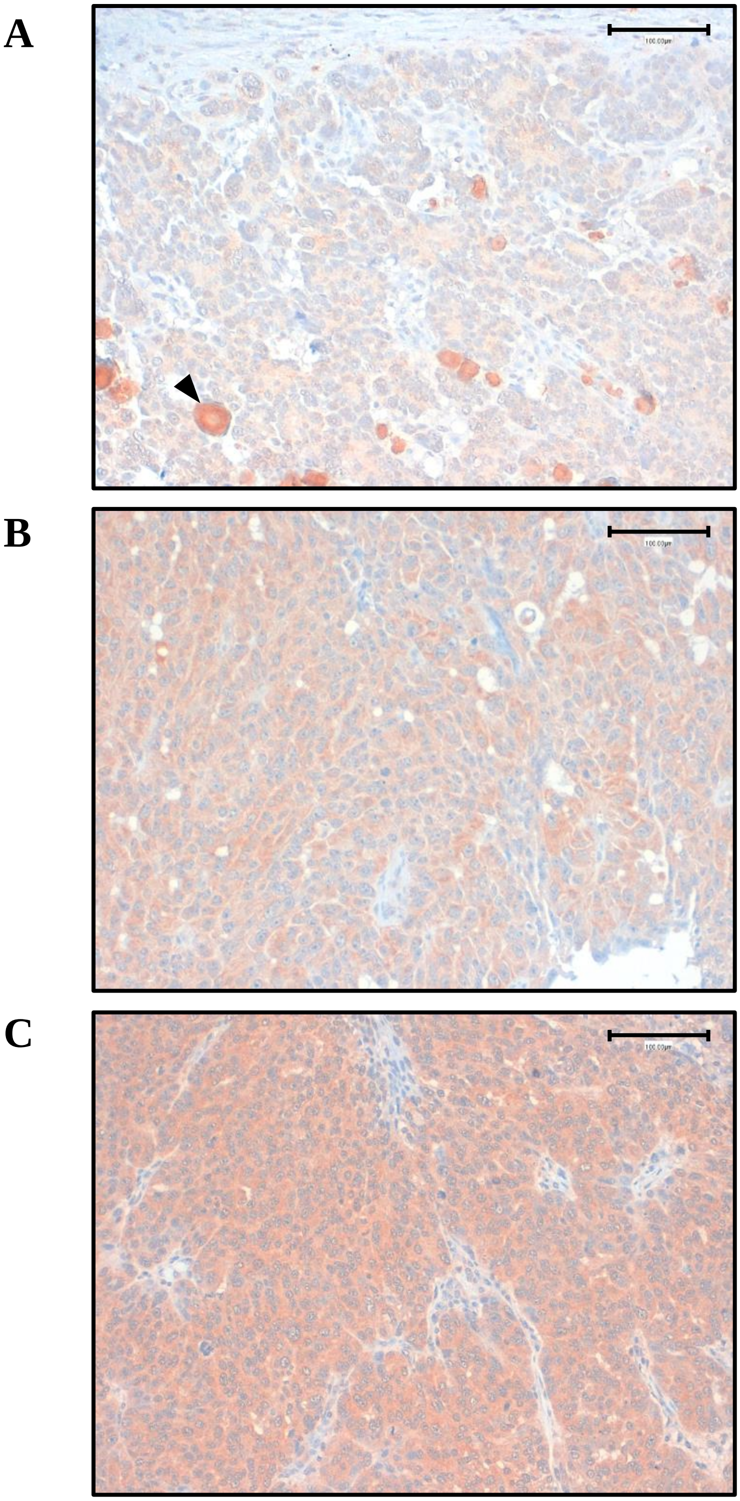

S1 Fig. Immunohistochemical staining examples of CA XII expression in high-grade serous ovarian carcinomas. Areas of predominantly weak (A), moderate (B), and strong (C) staining intensity (×200 magnification, scale bars: 100 µm). The arrowhead exemplarily points to one of the co-stained psammoma bodies in panel 'A'.
