# Supplementary material for: Carbonic anhydrase XII as biomarker and therapeutic target in ovarian carcinomas
Source: PLoS One. 2022 Jul 28;17(7):e0271630. doi: 10.1371/journal.pone.0271630 (PMC9333239; doi:10.1371/journal.pone.0271630)
Supplement: S1 Table — (PDF) [file pone.0271630.s002.pdf]

S1 Table. Minimal data set.sav

	Cohort	Histology	Grading
1	Ovarian carcinoma	endometrioid	G2
2	Ovarian carcinoma	serous high-grade	G3
3	Ovarian carcinoma	serous high-grade	G3
4	Ovarian carcinoma	serous high-grade	G3
5	Ovarian carcinoma	serous high-grade	G3
6	Ovarian carcinoma	serous high-grade	G3
7	Ovarian carcinoma	serous high-grade	G3
8	Ovarian carcinoma	serous high-grade	G3
9	Ovarian carcinoma	serous high-grade	G3
10	Ovarian carcinoma	clear cell	G3
11	Ovarian carcinoma	clear cell	G3
12	Ovarian carcinoma	serous low-grade	G1
13	Ovarian carcinoma	endometrioid	G1
14	Ovarian carcinoma	endometrioid	G1
15	Ovarian carcinoma	serous high-grade	G3
16	Ovarian carcinoma	serous high-grade	G3
17	Ovarian carcinoma	serous high-grade	G3
18	Ovarian carcinoma	serous high-grade	G3
19	Ovarian carcinoma	serous high-grade	G3
20	Ovarian carcinoma	serous high-grade	G3
21	Ovarian carcinoma	serous high-grade	G3
22	Ovarian carcinoma	serous high-grade	G3
23	Ovarian carcinoma	serous high-grade	G3
24	Ovarian carcinoma	endometrioid	G3
25	Ovarian carcinoma	endometrioid	GX/missing
26	Ovarian carcinoma	serous high-grade	G3
27	Ovarian carcinoma	serous high-grade	G3
28	Ovarian carcinoma	serous high-grade	G3
29	Ovarian carcinoma	clear cell	G3
30	Ovarian carcinoma	endometrioid	G3
31	Ovarian carcinoma	missing	GX/missing
32	Ovarian carcinoma	mucinous	GX/missing
33	Ovarian carcinoma	serous low-grade	G1
34	Ovarian carcinoma	serous low-grade	G1
35	Ovarian carcinoma	serous low-grade	G1
36	Ovarian carcinoma	serous low-grade	G1
37	Ovarian carcinoma	endometrioid	G2

S1 Table. Minimal data set.sav

	FIGO	Primary_tumor_expansion	Nodal_status	Distant_metastasis
1	FIGO III	T3	N1	MX/missing
2	FIGO III	T3	NX/missing	MX/missing
3	FIGO III	T3	NX/missing	MX/missing
4	FIGO III	T3	NX/missing	MX/missing
5	FIGO III	T3	NX/missing	MX/missing
6	FIGO III	T3	NX/missing	MX/missing
7	FIGO III	T3	NX/missing	MX/missing
8	FIGO III	T3	N1	MX/missing
9	FIGO III	T2	N1	MX/missing
10	FIGO I	T1	N0	MX/missing
11	FIGO I	T1	NX/missing	MX/missing
12	FIGO II	T2	NX/missing	MX/missing
13	FIGO II	T2	N0	MX/missing
14	FIGO IV	TX/missing	NX/missing	M1
15	FIGO III	T3	NX/missing	MX/missing
16	FIGO III	T3	NX/missing	MX/missing
17	FIGO III	T3	N0	MX/missing
18	FIGO III	T3	NX/missing	MX/missing
19	FIGO III	T1	N1	MX/missing
20	FIGO III	T3	N1	MX/missing
21	FIGO III	T3	NX/missing	MX/missing
22	FIGO III	T3	NX/missing	MX/missing
23	FIGO III	T3	N1	MX/missing
24	FIGO III	T3	NX/missing	MX/missing
25	missing	TX/missing	NX/missing	MX/missing
26	FIGO IV	T3	N1	M1
27	FIGO III	T3	N1	MX/missing
28	FIGO III	T2	N1	MX/missing
29	FIGO III	T2	N1	MX/missing
30	FIGO III	T3	NX/missing	MX/missing
31	FIGO III	T3	N1	MX/missing
32	FIGO I	T1	N0	M0
33	FIGO III	T3	N0	MX/missing
34	FIGO I	T1	NX/missing	MX/missing
35	FIGO III	T1	N1	MX/missing
36	FIGO III	T3	N1	MX/missing
37	FIGO I	T1	N0	MX/missing

S1 Table. Minimal data set.sav

	Age	Grouped_age	Median_age_HGSC	Death
1	55	50-59	not applicable	living
2	68	60-69	> median age	dead
3	74	70-79	> median age	dead
4	64	60-69	> median age	dead
5	60	60-69	< median age	dead
6	63	60-69	> median age	dead
7	51	50-59	< median age	dead
8	33	30-39	< median age	living
9	54	50-59	< median age	dead
10	51	50-59	not applicable	dead
11	56	50-59	not applicable	living
12	82	80-89	not applicable	dead
13	51	50-59	not applicable	dead
14	50	50-59	not applicable	living
15	74	70-79	> median age	dead
16	73	70-79	> median age	dead
17	64	60-69	> median age	living
18	64	60-69	> median age	dead
19	56	50-59	< median age	dead
20	63	60-69	> median age	dead
21	82	80-89	> median age	dead
22	78	70-79	> median age	dead
23	76	70-79	> median age	dead
24	71	70-79	not applicable	living
25	56	50-59	not applicable	dead
26	43	40-49	< median age	living
27	64	60-69	> median age	dead
28	62	60-69	< median age	dead
29	44	40-49	not applicable	dead
30	72	70-79	not applicable	dead
31	78	70-79	not applicable	dead
32	49	40-49	not applicable	living
33	54	50-59	not applicable	living
34	41	40-49	not applicable	missing
35	40	40-49	not applicable	living
36	48	40-49	not applicable	living
37	51	50-59	not applicable	dead

S1 Table. Minimal data set.sav

	Survival_months	Survival_years	Predominant_staining_intensity
1	201,12	16,76	moderate staining
2	24,96	2,08	moderate staining
3	14,16	1,18	strong staining
4	2,16	,18	moderate staining
5	33,12	2,76	strong staining
6	36,96	3,08	moderate staining
7	9,24	,77	strong staining
8	25,44	2,12	moderate staining
9	10,08	,84	moderate staining
10	62,88	5,24	weak staining
11	230,52	19,21	moderate staining
12	161,88	13,49	moderate staining
13	80,40	6,70	weak staining
14	13,80	1,15	moderate staining
15	23,76	1,98	weak staining
16	4,80	,40	moderate staining
17	,48	,04	moderate staining
18	3,72	,31	strong staining
19	81,12	6,76	moderate staining
20	28,80	2,40	strong staining
21	17,28	1,44	strong staining
22	8,40	,70	moderate staining
23	15,84	1,32	strong staining
24	,00	,00	moderate staining
25	105,12	8,76	moderate staining
26	15,48	1,29	strong staining
27	30,60	2,55	strong staining
28	12,60	1,05	moderate staining
29	17,04	1,42	moderate staining
30	82,80	6,90	moderate staining
31	88,56	7,38	strong staining
32	184,92	15,41	moderate staining
33	211,68	17,64	weak staining
34	missing	missing	strong staining
35	81,48	6,79	weak staining
36	166,20	13,85	moderate staining
37	35,04	2,92	moderate staining

S1 Table. Minimal data set.sav

	Percentage_of_positive_stained_cells	Immunoreactive_score
1	>80%	8
2	51-80%	6
3	10-50%	6
4	51-80%	6
5	51-80%	9
6	>80%	8
7	>80%	12
8	51-80%	6
9	>80%	8
10	>80%	4
11	>80%	8
12	>80%	8
13	>80%	4
14	>80%	8
15	>80%	4
16	>80%	8
17	51-80%	6
18	>80%	12
19	>80%	8
20	>80%	12
21	>80%	12
22	>80%	8
23	>80%	12
24	>80%	8
25	>80%	8
26	>80%	12
27	>80%	12
28	>80%	8
29	>80%	8
30	>80%	8
31	>80%	12
32	>80%	8
33	51-80%	3
34	51-80%	9
35	>80%	4
36	>80%	8
37	>80%	8

S1 Table. Minimal data set.sav

	Positive_IRS	Cutoff_IRS	Grouped_IRS
1	IRS >2	IRS ≤8	IRS 3-8
2	IRS >2	IRS ≤8	IRS 3-8
3	IRS >2	IRS ≤8	IRS 3-8
4	IRS >2	IRS ≤8	IRS 3-8
5	IRS >2	IRS >8	IRS 9-12
6	IRS >2	IRS ≤8	IRS 3-8
7	IRS >2	IRS >8	IRS 9-12
8	IRS >2	IRS ≤8	IRS 3-8
9	IRS >2	IRS ≤8	IRS 3-8
10	IRS >2	IRS ≤8	IRS 3-8
11	IRS >2	IRS ≤8	IRS 3-8
12	IRS >2	IRS ≤8	IRS 3-8
13	IRS >2	IRS ≤8	IRS 3-8
14	IRS >2	IRS ≤8	IRS 3-8
15	IRS >2	IRS ≤8	IRS 3-8
16	IRS >2	IRS ≤8	IRS 3-8
17	IRS >2	IRS ≤8	IRS 3-8
18	IRS >2	IRS >8	IRS 9-12
19	IRS >2	IRS ≤8	IRS 3-8
20	IRS >2	IRS >8	IRS 9-12
21	IRS >2	IRS >8	IRS 9-12
22	IRS >2	IRS ≤8	IRS 3-8
23	IRS >2	IRS >8	IRS 9-12
24	IRS >2	IRS ≤8	IRS 3-8
25	IRS >2	IRS ≤8	IRS 3-8
26	IRS >2	IRS >8	IRS 9-12
27	IRS >2	IRS >8	IRS 9-12
28	IRS >2	IRS ≤8	IRS 3-8
29	IRS >2	IRS ≤8	IRS 3-8
30	IRS >2	IRS ≤8	IRS 3-8
31	IRS >2	IRS >8	IRS 9-12
32	IRS >2	IRS ≤8	IRS 3-8
33	IRS >2	IRS ≤8	IRS 3-8
34	IRS >2	IRS >8	IRS 9-12
35	IRS >2	IRS ≤8	IRS 3-8
36	IRS >2	IRS ≤8	IRS 3-8
37	IRS >2	IRS ≤8	IRS 3-8

S1 Table. Minimal data set.sav

	Presence_of_strong_SI	Percentage_of_strong_SI
1	areas of strong staining intensity	<10%
2	areas of strong staining intensity	<10%
3	areas of strong staining intensity	10-50%
4	areas of strong staining intensity	<10%
5	areas of strong staining intensity	51-80%
6	areas of strong staining intensity	10-50%
7	areas of strong staining intensity	51-80%
8	areas of strong staining intensity	10-50%
9	areas of strong staining intensity	<10%
10	areas of strong staining intensity	<10%
11	areas of strong staining intensity	<10%
12	areas of strong staining intensity	10-50%
13	no areas of strong staining intensity	no strong staining intensity
14	areas of strong staining intensity	<10%
15	areas of strong staining intensity	<10%
16	areas of strong staining intensity	<10%
17	areas of strong staining intensity	<10%
18	areas of strong staining intensity	51-80%
19	areas of strong staining intensity	10-50%
20	areas of strong staining intensity	>80%
21	areas of strong staining intensity	51-80%
22	areas of strong staining intensity	10-50%
23	areas of strong staining intensity	51-80%
24	areas of strong staining intensity	10-50%
25	areas of strong staining intensity	10-50%
26	areas of strong staining intensity	51-80%
27	areas of strong staining intensity	51-80%
28	areas of strong staining intensity	<10%
29	areas of strong staining intensity	10-50%
30	areas of strong staining intensity	10-50%
31	areas of strong staining intensity	51-80%
32	areas of strong staining intensity	<10%
33	areas of strong staining intensity	<10%
34	areas of strong staining intensity	10-50%
35	areas of strong staining intensity	<10%
36	areas of strong staining intensity	<10%
37	areas of strong staining intensity	<10%

S1 Table. Minimal data set.sav

	Cohort	Histology	Grading
38	Ovarian carcinoma	endometrioid	G2
39	Ovarian carcinoma	mucinous	G2
40	Ovarian carcinoma	serous high-grade	G3
41	Ovarian carcinoma	serous high-grade	G3
42	Ovarian carcinoma	serous high-grade	G3
43	Ovarian carcinoma	serous high-grade	G3
44	Ovarian carcinoma	serous high-grade	G3
45	Ovarian carcinoma	clear cell	G3
46	Ovarian carcinoma	clear cell	G3
47	Ovarian carcinoma	serous low-grade	G1
48	Ovarian carcinoma	serous low-grade	G1
49	Ovarian carcinoma	serous high-grade	G3
50	Ovarian carcinoma	serous high-grade	G3
51	Ovarian carcinoma	clear cell	G3
52	Ovarian carcinoma	clear cell	G3
53	Ovarian carcinoma	endometrioid	G3
54	Ovarian carcinoma	missing	GX/missing
55	Ovarian carcinoma	serous low-grade	G1
56	Ovarian carcinoma	serous low-grade	G1
57	Ovarian carcinoma	endometrioid	G2
58	Ovarian carcinoma	serous high-grade	G3
59	Ovarian carcinoma	serous high-grade	G3
60	Ovarian carcinoma	serous high-grade	G3
61	Ovarian carcinoma	serous high-grade	G3
62	Ovarian carcinoma	endometrioid	G3
63	Ovarian carcinoma	missing	GX/missing
64	Ovarian carcinoma	serous low-grade	G1
65	Ovarian carcinoma	serous low-grade	G1
66	Ovarian carcinoma	endometrioid	G1
67	Ovarian carcinoma	mucinous	G1
68	Ovarian carcinoma	serous high-grade	G3
69	Ovarian carcinoma	serous high-grade	G3
70	Ovarian carcinoma	serous high-grade	G3
71	Ovarian carcinoma	serous high-grade	G3
72	Ovarian carcinoma	serous high-grade	G3
73	Ovarian carcinoma	clear cell	G3
74	Ovarian carcinoma	endometrioid	G3

S1 Table. Minimal data set.sav

	FIGO	Primary_tumor_expansion	Nodal_status	Distant_metastasis
38	FIGO I	T1	NX/missing	MX/missing
39	FIGO I	T1	N0	MX/missing
40	FIGO III	T3	N1	MX/missing
41	FIGO III	T3	N0	MX/missing
42	FIGO III	T3	NX/missing	MX/missing
43	FIGO III	T3	N1	MX/missing
44	FIGO III	T3	N1	MX/missing
45	FIGO I	T1	N0	MX/missing
46	FIGO III	T1	N1	MX/missing
47	FIGO III	T3	N1	MX/missing
48	FIGO I	T1	NX/missing	MX/missing
49	FIGO III	T3	NX/missing	MX/missing
50	FIGO III	TX/missing	NX/missing	MX/missing
51	FIGO III	T2	N1	MX/missing
52	FIGO III	T2	N1	MX/missing
53	FIGO III	T3	NX/missing	MX/missing
54	FIGO I	T1	N0	MX/missing
55	FIGO II	T2	NX/missing	MX/missing
56	FIGO I	T1	NX/missing	MX/missing
57	FIGO III	T3	N0	MX/missing
58	FIGO III	T3	NX/missing	MX/missing
59	FIGO III	T3	NX/missing	MX/missing
60	FIGO III	T3	NX/missing	MX/missing
61	FIGO IV	TX/missing	N1	M1
62	FIGO III	T3	NX/missing	MX/missing
63	FIGO I	T1	N0	MX/missing
64	FIGO III	T1	N1	MX/missing
65	FIGO I	T1	N0	MX/missing
66	FIGO I	T1	N0	MX/missing
67	FIGO II	T2	N0	MX/missing
68	FIGO III	T3	NX/missing	MX/missing
69	FIGO III	T3	NX/missing	MX/missing
70	FIGO III	T3	NX/missing	MX/missing
71	FIGO III	T3	NX/missing	MX/missing
72	FIGO III	T3	NX/missing	MX/missing
73	FIGO III	T3	N1	MX/missing
74	FIGO III	T3	N1	MX/missing

S1 Table. Minimal data set.sav

	Age	Grouped_age	Median_age_HGSC	Death
38	78	70-79	not applicable	dead
39	58	50-59	not applicable	living
40	53	50-59	< median age	dead
41	62	60-69	< median age	dead
42	43	40-49	< median age	dead
43	47	40-49	< median age	dead
44	48	40-49	< median age	dead
45	54	50-59	not applicable	living
46	52	50-59	not applicable	dead
47	36	30-39	not applicable	dead
48	37	30-39	not applicable	living
49	77	70-79	> median age	dead
50	50	50-59	< median age	dead
51	58	50-59	not applicable	dead
52	50	50-59	not applicable	dead
53	68	60-69	not applicable	dead
54	56	50-59	not applicable	living
55	45	40-49	not applicable	living
56	32	30-39	not applicable	dead
57	65	60-69	not applicable	living
58	62	60-69	< median age	dead
59	66	60-69	> median age	dead
60	70	70-79	> median age	dead
61	57	50-59	< median age	dead
62	67	60-69	not applicable	dead
63	65	60-69	not applicable	dead
64	49	40-49	not applicable	dead
65	54	50-59	not applicable	living
66	49	40-49	not applicable	living
67	44	40-49	not applicable	living
68	76	70-79	> median age	dead
69	57	50-59	< median age	dead
70	60	60-69	< median age	dead
71	49	40-49	< median age	dead
72	83	80-89	> median age	dead
73	72	70-79	not applicable	living
74	61	60-69	not applicable	dead

S1 Table. Minimal data set.sav

	Survival_months	Survival_years	Predominant_staining_intensity
38	27,36	2,28	moderate staining
39	,24	,02	moderate staining
40	94,08	7,84	strong staining
41	10,80	,90	moderate staining
42	15,24	1,27	moderate staining
43	35,16	2,93	strong staining
44	24,00	2,00	strong staining
45	167,64	13,97	moderate staining
46	32,52	2,71	weak staining
47	109,68	9,14	moderate staining
48	162,36	13,53	weak staining
49	27,00	2,25	moderate staining
50	44,76	3,73	strong staining
51	19,80	1,65	moderate staining
52	3,48	,29	weak staining
53	33,24	2,77	weak staining
54	187,08	15,59	strong staining
55	146,64	12,22	moderate staining
56	15,60	1,30	weak staining
57	175,20	14,60	strong staining
58	26,04	2,17	strong staining
59	5,28	,44	strong staining
60	9,48	,79	strong staining
61	15,72	1,31	moderate staining
62	15,12	1,26	weak staining
63	50,88	4,24	moderate staining
64	14,40	1,20	moderate staining
65	136,68	11,39	weak staining
66	171,12	14,26	moderate staining
67	133,44	11,12	moderate staining
68	5,40	,45	moderate staining
69	84,60	7,05	weak staining
70	8,88	,74	moderate staining
71	30,60	2,55	moderate staining
72	12,60	1,05	moderate staining
73	167,40	13,95	strong staining
74	14,16	1,18	strong staining

	Percentage_of_positive_stained_cells	Immunoreactive_score
38	51-80%	6
39	>80%	8
40	>80%	12
41	>80%	8
42	>80%	8
43	>80%	12
44	>80%	12
45	>80%	8
46	>80%	4
47	51-80%	6
48	51-80%	3
49	51-80%	6
50	>80%	12
51	>80%	8
52	>80%	4
53	>80%	4
54	>80%	12
55	51-80%	6
56	>80%	4
57	>80%	12
58	>80%	12
59	>80%	12
60	>80%	12
61	>80%	8
62	>80%	4
63	>80%	8
64	>80%	8
65	>80%	4
66	>80%	8
67	10-50%	4
68	>80%	8
69	>80%	4
70	>80%	8
71	>80%	8
72	>80%	8
73	>80%	12
74	>80%	12

S1 Table. Minimal data set.sav

	Positive_IRS	Cutoff_IRS	Grouped_IRS
38	IRS >2	IRS ≤8	IRS 3-8
39	IRS >2	IRS ≤8	IRS 3-8
40	IRS >2	IRS >8	IRS 9-12
41	IRS >2	IRS ≤8	IRS 3-8
42	IRS >2	IRS ≤8	IRS 3-8
43	IRS >2	IRS >8	IRS 9-12
44	IRS >2	IRS >8	IRS 9-12
45	IRS >2	IRS ≤8	IRS 3-8
46	IRS >2	IRS ≤8	IRS 3-8
47	IRS >2	IRS ≤8	IRS 3-8
48	IRS >2	IRS ≤8	IRS 3-8
49	IRS >2	IRS ≤8	IRS 3-8
50	IRS >2	IRS >8	IRS 9-12
51	IRS >2	IRS ≤8	IRS 3-8
52	IRS >2	IRS ≤8	IRS 3-8
53	IRS >2	IRS ≤8	IRS 3-8
54	IRS >2	IRS >8	IRS 9-12
55	IRS >2	IRS ≤8	IRS 3-8
56	IRS >2	IRS ≤8	IRS 3-8
57	IRS >2	IRS >8	IRS 9-12
58	IRS >2	IRS >8	IRS 9-12
59	IRS >2	IRS >8	IRS 9-12
60	IRS >2	IRS >8	IRS 9-12
61	IRS >2	IRS ≤8	IRS 3-8
62	IRS >2	IRS ≤8	IRS 3-8
63	IRS >2	IRS ≤8	IRS 3-8
64	IRS >2	IRS ≤8	IRS 3-8
65	IRS >2	IRS ≤8	IRS 3-8
66	IRS >2	IRS ≤8	IRS 3-8
67	IRS >2	IRS ≤8	IRS 3-8
68	IRS >2	IRS ≤8	IRS 3-8
69	IRS >2	IRS ≤8	IRS 3-8
70	IRS >2	IRS ≤8	IRS 3-8
71	IRS >2	IRS ≤8	IRS 3-8
72	IRS >2	IRS ≤8	IRS 3-8
73	IRS >2	IRS >8	IRS 9-12
74	IRS >2	IRS >8	IRS 9-12

S1 Table. Minimal data set.sav

	Presence_of_strong_SI	Percentage_of_strong_SI
38	areas of strong staining intensity	<10%
39	areas of strong staining intensity	10-50%
40	areas of strong staining intensity	>80%
41	areas of strong staining intensity	10-50%
42	areas of strong staining intensity	<10%
43	areas of strong staining intensity	>80%
44	areas of strong staining intensity	>80%
45	areas of strong staining intensity	<10%
46	areas of strong staining intensity	<10%
47	areas of strong staining intensity	<10%
48	areas of strong staining intensity	<10%
49	areas of strong staining intensity	<10%
50	areas of strong staining intensity	51-80%
51	areas of strong staining intensity	<10%
52	areas of strong staining intensity	<10%
53	areas of strong staining intensity	10-50%
54	areas of strong staining intensity	10-50%
55	areas of strong staining intensity	<10%
56	areas of strong staining intensity	<10%
57	areas of strong staining intensity	51-80%
58	areas of strong staining intensity	>80%
59	areas of strong staining intensity	51-80%
60	areas of strong staining intensity	>80%
61	areas of strong staining intensity	10-50%
62	areas of strong staining intensity	<10%
63	areas of strong staining intensity	10-50%
64	areas of strong staining intensity	10-50%
65	areas of strong staining intensity	<10%
66	areas of strong staining intensity	<10%
67	areas of strong staining intensity	<10%
68	areas of strong staining intensity	10-50%
69	areas of strong staining intensity	<10%
70	areas of strong staining intensity	10-50%
71	areas of strong staining intensity	<10%
72	no areas of strong staining intensity	no strong staining intensity
73	areas of strong staining intensity	51-80%
74	areas of strong staining intensity	51-80%

S1 Table. Minimal data set.sav

	Cohort	Histology	Grading
75	Ovarian carcinoma	serous low-grade	G1
76	Ovarian carcinoma	serous low-grade	G1
77	Ovarian carcinoma	serous low-grade	G1
78	Ovarian carcinoma	endometrioid	G1
79	Ovarian carcinoma	mucinous	G2
80	Ovarian carcinoma	mucinous	G2
81	Ovarian carcinoma	mucinous	G2
82	Ovarian carcinoma	serous high-grade	G3
83	Ovarian carcinoma	serous high-grade	G3
84	Ovarian carcinoma	serous high-grade	G3
85	Ovarian carcinoma	serous high-grade	G3
86	Ovarian carcinoma	serous high-grade	G3
87	Ovarian carcinoma	clear cell	G3
88	Ovarian carcinoma	serous low-grade	G1
89	Ovarian carcinoma	serous low-grade	G1
90	Ovarian carcinoma	serous high-grade	G3
91	Ovarian carcinoma	serous high-grade	G3
92	Ovarian carcinoma	serous high-grade	G3
93	Ovarian carcinoma	serous high-grade	G3
94	Ovarian carcinoma	serous high-grade	G3
95	Ovarian carcinoma	serous high-grade	G3
96	Ovarian carcinoma	serous high-grade	G3
97	Ovarian carcinoma	serous high-grade	G3
98	Ovarian carcinoma	serous high-grade	G3
99	Ovarian carcinoma	serous high-grade	G3
100	Ovarian carcinoma	clear cell	G3
101	Ovarian carcinoma	endometrioid	G3
102	Ovarian carcinoma	missing	GX/missing
103	Ovarian carcinoma	serous low-grade	G1
104	Ovarian carcinoma	mucinous	G1
105	Ovarian carcinoma	mucinous	G1
106	Ovarian carcinoma	endometrioid	G2
107	Ovarian carcinoma	serous high-grade	G3
108	Ovarian carcinoma	serous high-grade	G3
109	Ovarian carcinoma	serous high-grade	G3
110	Ovarian carcinoma	serous high-grade	G3
111	Ovarian carcinoma	serous high-grade	G3

S1 Table. Minimal data set.sav

	FIGO	Primary_tumor_expansion	Nodal_status	Distant_metastasis
75	FIGO I	T1	N0	MX/missing
76	FIGO I	T1	NX/missing	MX/missing
77	FIGO III	T3	N0	MX/missing
78	FIGO I	T1	N0	MX/missing
79	FIGO I	T1	N0	MX/missing
80	FIGO I	T2	N0	MX/missing
81	FIGO I	T1	NX/missing	MX/missing
82	FIGO III	T3	NX/missing	MX/missing
83	FIGO III	T3	N1	MX/missing
84	FIGO III	T3	N1	MX/missing
85	FIGO III	T3	N1	MX/missing
86	FIGO III	T3	N0	MX/missing
87	missing	T3	NX/missing	MX/missing
88	FIGO III	T3	N1	MX/missing
89	FIGO I	T1	N0	MX/missing
90	FIGO III	T3	NX/missing	MX/missing
91	FIGO III	T3	N1	MX/missing
92	FIGO III	T2	N1	MX/missing
93	FIGO III	T3	N1	MX/missing
94	FIGO III	T3	N1	MX/missing
95	FIGO III	T3	N1	MX/missing
96	FIGO III	T3	NX/missing	MX/missing
97	FIGO III	T3	N1	MX/missing
98	FIGO III	T3	N1	MX/missing
99	FIGO III	T3	N1	MX/missing
100	FIGO I	T1	NX/missing	MX/missing
101	FIGO III	T3	N1	MX/missing
102	FIGO IV	T3	NX/missing	M1
103	FIGO III	T3	N1	MX/missing
104	FIGO III	T3	N0	MX/missing
105	FIGO III	T3	N1	MX/missing
106	FIGO I	T1	NX/missing	MX/missing
107	FIGO III	T3	NX/missing	MX/missing
108	FIGO III	T3	NX/missing	MX/missing
109	FIGO I	T1	N0	MX/missing
110	FIGO III	T3	N1	MX/missing
111	FIGO II	T2	NX/missing	MX/missing

	Age	Grouped_age	Median_age_HGSC	Death
75	50	50-59	not applicable	living
76	51	50-59	not applicable	living
77	42	40-49	not applicable	living
78	64	60-69	not applicable	living
79	66	60-69	not applicable	living
80	42	40-49	not applicable	living
81	30	30-39	not applicable	living
82	75	70-79	> median age	dead
83	45	40-49	< median age	dead
84	46	40-49	< median age	dead
85	46	40-49	< median age	dead
86	50	50-59	< median age	living
87	42	40-49	not applicable	dead
88	36	30-39	not applicable	dead
89	51	50-59	not applicable	living
90	77	70-79	> median age	dead
91	65	60-69	> median age	dead
92	57	50-59	< median age	dead
93	58	50-59	< median age	dead
94	52	50-59	< median age	dead
95	74	70-79	> median age	dead
96	59	50-59	< median age	dead
97	44	40-49	< median age	dead
98	58	50-59	< median age	dead
99	73	70-79	> median age	dead
100	43	40-49	not applicable	living
101	57	50-59	not applicable	dead
102	36	30-39	not applicable	dead
103	47	40-49	not applicable	living
104	31	30-39	not applicable	dead
105	40	40-49	not applicable	dead
106	missing	missing	not applicable	dead
107	65	60-69	> median age	dead
108	78	70-79	> median age	dead
109	60	60-69	< median age	living
110	71	70-79	> median age	dead
111	88	80-89	> median age	dead

S1 Table. Minimal data set.sav

	Survival_months	Survival_years	Predominant_staining_intensity
75	164,16	13,68	moderate staining
76	126,72	10,56	moderate staining
77	2,16	,18	moderate staining
78	121,80	10,15	moderate staining
79	112,44	9,37	weak staining
80	119,76	9,98	weak staining
81	153,12	12,76	strong staining
82	4,56	,38	strong staining
83	83,40	6,95	moderate staining
84	30,96	2,58	moderate staining
85	25,68	2,14	moderate staining
86	150,96	12,58	weak staining
87	2,76	,23	weak staining
88	153,48	12,79	moderate staining
89	106,68	8,89	moderate staining
90	29,28	2,44	moderate staining
91	12,24	1,02	weak staining
92	43,56	3,63	moderate staining
93	29,88	2,49	moderate staining
94	18,72	1,56	weak staining
95	16,80	1,40	moderate staining
96	7,32	,61	weak staining
97	20,04	1,67	moderate staining
98	83,16	6,93	strong staining
99	45,00	3,75	strong staining
100	145,92	12,16	moderate staining
101	6,72	,56	moderate staining
102	74,52	6,21	weak staining
103	98,04	8,17	moderate staining
104	34,44	2,87	weak staining
105	27,36	2,28	weak staining
106	16,32	1,36	moderate staining
107	22,80	1,90	weak staining
108	50,64	4,22	weak staining
109	103,08	8,59	strong staining
110	50,64	4,22	strong staining
111	6,36	,53	weak staining

	Percentage_of_positive_stained_cells	Immunoreactive_score
75	51-80%	6
76	>80%	8
77	>80%	8
78	>80%	8
79	>80%	4
80	>80%	4
81	>80%	12
82	>80%	12
83	>80%	8
84	>80%	8
85	>80%	8
86	51-80%	3
87	>80%	4
88	>80%	8
89	>80%	8
90	>80%	8
91	51-80%	3
92	>80%	8
93	>80%	8
94	51-80%	3
95	51-80%	6
96	>80%	4
97	>80%	8
98	>80%	12
99	>80%	12
100	>80%	8
101	>80%	8
102	51-80%	3
103	>80%	8
104	>80%	4
105	<10%	1
106	>80%	8
107	>80%	4
108	>80%	4
109	>80%	12
110	>80%	12
111	>80%	4

	Positive_IRS	Cutoff_IRS	Grouped_IRS
75	IRS >2	IRS ≤8	IRS 3-8
76	IRS >2	IRS ≤8	IRS 3-8
77	IRS >2	IRS ≤8	IRS 3-8
78	IRS >2	IRS ≤8	IRS 3-8
79	IRS >2	IRS ≤8	IRS 3-8
80	IRS >2	IRS ≤8	IRS 3-8
81	IRS >2	IRS >8	IRS 9-12
82	IRS >2	IRS >8	IRS 9-12
83	IRS >2	IRS ≤8	IRS 3-8
84	IRS >2	IRS ≤8	IRS 3-8
85	IRS >2	IRS ≤8	IRS 3-8
86	IRS >2	IRS ≤8	IRS 3-8
87	IRS >2	IRS ≤8	IRS 3-8
88	IRS >2	IRS ≤8	IRS 3-8
89	IRS >2	IRS ≤8	IRS 3-8
90	IRS >2	IRS ≤8	IRS 3-8
91	IRS >2	IRS ≤8	IRS 3-8
92	IRS >2	IRS ≤8	IRS 3-8
93	IRS >2	IRS ≤8	IRS 3-8
94	IRS >2	IRS ≤8	IRS 3-8
95	IRS >2	IRS ≤8	IRS 3-8
96	IRS >2	IRS ≤8	IRS 3-8
97	IRS >2	IRS ≤8	IRS 3-8
98	IRS >2	IRS >8	IRS 9-12
99	IRS >2	IRS >8	IRS 9-12
100	IRS >2	IRS ≤8	IRS 3-8
101	IRS >2	IRS ≤8	IRS 3-8
102	IRS >2	IRS ≤8	IRS 3-8
103	IRS >2	IRS ≤8	IRS 3-8
104	IRS >2	IRS ≤8	IRS 3-8
105	IRS ≤2	IRS ≤8	IRS 0-2
106	IRS >2	IRS ≤8	IRS 3-8
107	IRS >2	IRS ≤8	IRS 3-8
108	IRS >2	IRS ≤8	IRS 3-8
109	IRS >2	IRS >8	IRS 9-12
110	IRS >2	IRS >8	IRS 9-12
111	IRS >2	IRS ≤8	IRS 3-8

	Presence_of_strong_SI	Percentage_of_strong_SI
75	areas of strong staining intensity	<10%
76	areas of strong staining intensity	<10%
77	areas of strong staining intensity	10-50%
78	areas of strong staining intensity	<10%
79	areas of strong staining intensity	<10%
80	areas of strong staining intensity	<10%
81	areas of strong staining intensity	10-50%
82	areas of strong staining intensity	>80%
83	no areas of strong staining intensity	no strong staining intensity
84	areas of strong staining intensity	10-50%
85	areas of strong staining intensity	<10%
86	no areas of strong staining intensity	no strong staining intensity
87	areas of strong staining intensity	<10%
88	areas of strong staining intensity	10-50%
89	areas of strong staining intensity	<10%
90	no areas of strong staining intensity	no strong staining intensity
91	no areas of strong staining intensity	no strong staining intensity
92	areas of strong staining intensity	10-50%
93	areas of strong staining intensity	<10%
94	no areas of strong staining intensity	no strong staining intensity
95	no areas of strong staining intensity	no strong staining intensity
96	no areas of strong staining intensity	no strong staining intensity
97	areas of strong staining intensity	10-50%
98	areas of strong staining intensity	51-80%
99	areas of strong staining intensity	51-80%
100	areas of strong staining intensity	<10%
101	areas of strong staining intensity	10-50%
102	areas of strong staining intensity	<10%
103	areas of strong staining intensity	<10%
104	no areas of strong staining intensity	no strong staining intensity
105	no areas of strong staining intensity	no strong staining intensity
106	areas of strong staining intensity	<10%
107	areas of strong staining intensity	<10%
108	areas of strong staining intensity	<10%
109	areas of strong staining intensity	51-80%
110	areas of strong staining intensity	51-80%
111	areas of strong staining intensity	<10%

S1 Table. Minimal data set.sav

	Cohort	Histology	Grading
112	Ovarian carcinoma	clear cell	G3
113	Ovarian carcinoma	serous low-grade	G1
114	Ovarian carcinoma	serous low-grade	G1
115	Ovarian carcinoma	endometrioid	G1
116	Ovarian carcinoma	mucinous	G2
117	Ovarian carcinoma	serous high-grade	G3
118	Ovarian carcinoma	serous high-grade	G3
119	Ovarian carcinoma	serous high-grade	G3
120	Ovarian carcinoma	serous high-grade	G3
121	Ovarian carcinoma	serous high-grade	G3
122	Ovarian carcinoma	serous high-grade	G3
123	Ovarian carcinoma	endometrioid	G3
124	Ovarian carcinoma	endometrioid	G1
125	Ovarian carcinoma	serous high-grade	G3
126	Ovarian carcinoma	serous high-grade	G3
127	Ovarian carcinoma	serous high-grade	G3
128	Ovarian carcinoma	serous high-grade	G3
129	Ovarian carcinoma	serous high-grade	G3
130	Ovarian carcinoma	serous high-grade	G3
131	Ovarian carcinoma	serous high-grade	G3
132	Ovarian carcinoma	serous high-grade	G3
133	Ovarian carcinoma	serous high-grade	G3
134	Ovarian carcinoma	serous high-grade	G3
135	Ovarian carcinoma	serous high-grade	G3
136	Ovarian carcinoma	serous high-grade	G3
137	Ovarian carcinoma	serous high-grade	G3
138	Ovarian carcinoma	seromucinous	G1
139	Ovarian carcinoma	serous low-grade	G1
140	Ovarian carcinoma	serous low-grade	G1
141	Ovarian carcinoma	mucinous	G1
142	Ovarian carcinoma	endometrioid	G1
143	Ovarian carcinoma	endometrioid	G1
144	Ovarian carcinoma	serous low-grade	G1
145	Ovarian carcinoma	serous low-grade	G1
146	Ovarian carcinoma	serous low-grade	G1
147	Ovarian carcinoma	endometrioid	G2
148	Ovarian carcinoma	mucinous	G2

S1 Table. Minimal data set.sav

	FIGO	Primary_tumor_expansion	Nodal_status	Distant_metastasis
112	FIGO I	T1	NX/missing	MX/missing
113	FIGO III	T3	N1	MX/missing
114	FIGO III	T3	NX/missing	M0
115	FIGO I	T1	N0	MX/missing
116	FIGO II	T2	N0	MX/missing
117	FIGO III	T3	NX/missing	MX/missing
118	FIGO III	T3	N1	MX/missing
119	FIGO III	T3	N1	MX/missing
120	FIGO III	T3	NX/missing	MX/missing
121	FIGO III	T3	N0	MX/missing
122	FIGO III	T3	N1	MX/missing
123	FIGO II	T2	N0	MX/missing
124	FIGO I	T1	N0	MX/missing
125	FIGO I	T1	N0	MX/missing
126	FIGO III	T3	NX/missing	MX/missing
127	FIGO III	T3	N0	MX/missing
128	FIGO III	T3	N0	MX/missing
129	FIGO I	T1	N0	MX/missing
130	FIGO III	T3	NX/missing	MX/missing
131	FIGO III	T3	NX/missing	MX/missing
132	FIGO III	T3	N1	MX/missing
133	FIGO I	T1	N0	MX/missing
134	FIGO III	T3	N0	MX/missing
135	FIGO III	T3	NX/missing	M0
136	FIGO II	T2	N0	MX/missing
137	FIGO II	T2	N0	MX/missing
138	FIGO I	T1	N0	M0
139	FIGO II	T2	N0	MX/missing
140	FIGO III	T3	N1	MX/missing
141	FIGO I	T1	NX/missing	MX/missing
142	FIGO I	T1	N0	MX/missing
143	FIGO I	T1	NX/missing	MX/missing
144	FIGO III	T3	N1	MX/missing
145	FIGO III	T3	NX/missing	MX/missing
146	FIGO IV	TX/missing	NX/missing	M1
147	FIGO III	T3	N0	MX/missing
148	FIGO III	T3	N1	MX/missing

S1 Table. Minimal data set.sav

	Age	Grouped_age	Median_age_HGSC	Death
112	65	60-69	not applicable	dead
113	52	50-59	not applicable	dead
114	78	70-79	not applicable	living
115	60	60-69	not applicable	dead
116	77	70-79	not applicable	dead
117	66	60-69	> median age	dead
118	73	70-79	> median age	dead
119	65	60-69	> median age	dead
120	65	60-69	> median age	dead
121	61	60-69	< median age	living
122	50	50-59	< median age	dead
123	53	50-59	not applicable	living
124	66	60-69	not applicable	living
125	53	50-59	< median age	living
126	54	50-59	< median age	dead
127	65	60-69	> median age	living
128	70	70-79	> median age	living
129	42	40-49	< median age	dead
130	58	50-59	< median age	living
131	70	70-79	> median age	dead
132	65	60-69	> median age	dead
133	54	50-59	< median age	dead
134	65	60-69	> median age	living
135	79	70-79	> median age	dead
136	72	70-79	> median age	dead
137	61	60-69	< median age	living
138	33	30-39	not applicable	living
139	77	70-79	not applicable	living
140	53	50-59	not applicable	dead
141	67	60-69	not applicable	living
142	79	70-79	not applicable	dead
143	66	60-69	not applicable	living
144	62	60-69	not applicable	dead
145	69	60-69	not applicable	dead
146	80	80-89	not applicable	dead
147	51	50-59	not applicable	living
148	66	60-69	not applicable	dead

S1 Table. Minimal data set.sav

	Survival_months	Survival_years	Predominant_staining_intensity
112	61,32	5,11	moderate staining
113	61,20	5,10	moderate staining
114	118,08	9,84	weak staining
115	63,36	5,28	weak staining
116	52,32	4,36	weak staining
117	13,68	1,14	moderate staining
118	65,40	5,45	weak staining
119	102,36	8,53	weak staining
120	21,36	1,78	weak staining
121	84,36	7,03	moderate staining
122	16,20	1,35	weak staining
123	82,56	6,88	weak staining
124	70,56	5,88	weak staining
125	77,64	6,47	moderate staining
126	39,60	3,30	weak staining
127	110,88	9,24	moderate staining
128	74,28	6,19	moderate staining
129	58,80	4,90	moderate staining
130	15,36	1,28	moderate staining
131	12,00	1,00	weak staining
132	27,84	2,32	weak staining
133	59,88	4,99	moderate staining
134	71,52	5,96	moderate staining
135	2,40	,20	moderate staining
136	19,08	1,59	moderate staining
137	67,20	5,60	moderate staining
138	56,61	4,72	weak staining
139	127,56	10,63	weak staining
140	75,75	6,31	strong staining
141	123,16	10,26	strong staining
142	35,80	2,98	weak staining
143	121,84	10,15	moderate staining
144	18,84	1,57	moderate staining
145	29,72	2,48	moderate staining
146	20,38	1,70	moderate staining
147	55,07	4,59	moderate staining
148	13,58	1,13	weak staining

S1 Table. Minimal data set.sav

	Percentage_of_positive_stained_cells	Immunoreactive_score
112	>80%	8
113	51-80%	6
114	>80%	4
115	51-80%	3
116	>80%	4
117	>80%	8
118	>80%	4
119	51-80%	3
120	>80%	4
121	>80%	8
122	51-80%	3
123	51-80%	3
124	>80%	4
125	>80%	8
126	>80%	4
127	>80%	8
128	>80%	8
129	>80%	8
130	>80%	8
131	51-80%	3
132	>80%	4
133	>80%	8
134	>80%	8
135	>80%	8
136	10-50%	4
137	>80%	8
138	51-80%	3
139	>80%	4
140	51-80%	9
141	51-80%	9
142	10-50%	2
143	>80%	8
144	>80%	8
145	>80%	8
146	>80%	8
147	>80%	8
148	51-80%	3

	Positive_IRS	Cutoff_IRS	Grouped_IRS
112	IRS >2	IRS ≤8	IRS 3-8
113	IRS >2	IRS ≤8	IRS 3-8
114	IRS >2	IRS ≤8	IRS 3-8
115	IRS >2	IRS ≤8	IRS 3-8
116	IRS >2	IRS ≤8	IRS 3-8
117	IRS >2	IRS ≤8	IRS 3-8
118	IRS >2	IRS ≤8	IRS 3-8
119	IRS >2	IRS ≤8	IRS 3-8
120	IRS >2	IRS ≤8	IRS 3-8
121	IRS >2	IRS ≤8	IRS 3-8
122	IRS >2	IRS ≤8	IRS 3-8
123	IRS >2	IRS ≤8	IRS 3-8
124	IRS >2	IRS ≤8	IRS 3-8
125	IRS >2	IRS ≤8	IRS 3-8
126	IRS >2	IRS ≤8	IRS 3-8
127	IRS >2	IRS ≤8	IRS 3-8
128	IRS >2	IRS ≤8	IRS 3-8
129	IRS >2	IRS ≤8	IRS 3-8
130	IRS >2	IRS ≤8	IRS 3-8
131	IRS >2	IRS ≤8	IRS 3-8
132	IRS >2	IRS ≤8	IRS 3-8
133	IRS >2	IRS ≤8	IRS 3-8
134	IRS >2	IRS ≤8	IRS 3-8
135	IRS >2	IRS ≤8	IRS 3-8
136	IRS >2	IRS ≤8	IRS 3-8
137	IRS >2	IRS ≤8	IRS 3-8
138	IRS >2	IRS ≤8	IRS 3-8
139	IRS >2	IRS ≤8	IRS 3-8
140	IRS >2	IRS >8	IRS 9-12
141	IRS >2	IRS >8	IRS 9-12
142	IRS ≤2	IRS ≤8	IRS 0-2
143	IRS >2	IRS ≤8	IRS 3-8
144	IRS >2	IRS ≤8	IRS 3-8
145	IRS >2	IRS ≤8	IRS 3-8
146	IRS >2	IRS ≤8	IRS 3-8
147	IRS >2	IRS ≤8	IRS 3-8
148	IRS >2	IRS ≤8	IRS 3-8

S1 Table. Minimal data set.sav

	Presence_of_strong_SI	Percentage_of_strong_SI
112	areas of strong staining intensity	<10%
113	areas of strong staining intensity	<10%
114	no areas of strong staining intensity	no strong staining intensity
115	no areas of strong staining intensity	no strong staining intensity
116	no areas of strong staining intensity	no strong staining intensity
117	areas of strong staining intensity	<10%
118	areas of strong staining intensity	<10%
119	no areas of strong staining intensity	no strong staining intensity
120	no areas of strong staining intensity	no strong staining intensity
121	no areas of strong staining intensity	no strong staining intensity
122	no areas of strong staining intensity	no strong staining intensity
123	no areas of strong staining intensity	no strong staining intensity
124	areas of strong staining intensity	<10%
125	areas of strong staining intensity	<10%
126	no areas of strong staining intensity	no strong staining intensity
127	areas of strong staining intensity	10-50%
128	areas of strong staining intensity	<10%
129	no areas of strong staining intensity	no strong staining intensity
130	no areas of strong staining intensity	no strong staining intensity
131	no areas of strong staining intensity	no strong staining intensity
132	areas of strong staining intensity	<10%
133	no areas of strong staining intensity	no strong staining intensity
134	no areas of strong staining intensity	no strong staining intensity
135	areas of strong staining intensity	<10%
136	areas of strong staining intensity	<10%
137	areas of strong staining intensity	<10%
138	no areas of strong staining intensity	no strong staining intensity
139	areas of strong staining intensity	<10%
140	areas of strong staining intensity	51-80%
141	areas of strong staining intensity	10-50%
142	no areas of strong staining intensity	no strong staining intensity
143	areas of strong staining intensity	10-50%
144	areas of strong staining intensity	<10%
145	areas of strong staining intensity	10-50%
146	areas of strong staining intensity	<10%
147	areas of strong staining intensity	<10%
148	no areas of strong staining intensity	no strong staining intensity

S1 Table. Minimal data set.sav

	Cohort	Histology	Grading
149	Ovarian carcinoma	seromucinous	G2
150	Ovarian carcinoma	mucinous	G2
151	Ovarian carcinoma	endometrioid	G2
152	Ovarian carcinoma	endometrioid	G2
153	Ovarian carcinoma	serous high-grade	G3
154	Ovarian carcinoma	serous high-grade	G3
155	Ovarian carcinoma	serous high-grade	G3
156	Ovarian carcinoma	serous high-grade	G3
157	Ovarian carcinoma	serous high-grade	G3
158	Ovarian carcinoma	serous high-grade	G3
159	Ovarian carcinoma	serous high-grade	G3
160	Ovarian carcinoma	serous high-grade	G3
161	Ovarian carcinoma	serous high-grade	G3
162	Ovarian carcinoma	serous high-grade	G3
163	Ovarian carcinoma	serous high-grade	G3
164	Ovarian carcinoma	serous high-grade	G3
165	Ovarian carcinoma	serous high-grade	G3
166	Ovarian carcinoma	serous high-grade	G3
167	Ovarian carcinoma	serous high-grade	G3
168	Ovarian carcinoma	serous high-grade	G3
169	Ovarian carcinoma	serous high-grade	G3
170	Ovarian carcinoma	serous high-grade	G3
171	Ovarian carcinoma	serous high-grade	G3
172	Ovarian carcinoma	serous high-grade	G3
173	Ovarian carcinoma	undifferentiated	G3
174	Ovarian carcinoma	serous high-grade	G3
175	Ovarian carcinoma	endometrioid	G3
176	Ovarian carcinoma	serous high-grade	G3
177	Ovarian carcinoma	serous high-grade	G3
178	Ovarian carcinoma	serous high-grade	G3
179	Ovarian carcinoma	serous high-grade	G3
180	Ovarian carcinoma	serous high-grade	G3
181	Ovarian carcinoma	serous high-grade	G3
182	Ovarian carcinoma	serous high-grade	G3
183	Ovarian carcinoma	serous high-grade	G3
184	Ovarian carcinoma	serous high-grade	G3
185	Ovarian carcinoma	serous high-grade	G3

S1 Table. Minimal data set.sav

	FIGO	Primary_tumor_expansion	Nodal_status	Distant_metastasis
149	FIGO I	T1	N0	MX/missing
150	FIGO III	T1	N1	MX/missing
151	FIGO I	T1	NX/missing	MX/missing
152	FIGO II	T2	N0	M0
153	FIGO III	T3	N0	M0
154	FIGO IV	T3	NX/missing	M1
155	FIGO IV	T3	NX/missing	M1
156	FIGO IV	T1	NX/missing	M1
157	FIGO III	T3	NX/missing	M0
158	FIGO III	T3	NX/missing	MX/missing
159	FIGO III	T3	N1	MX/missing
160	FIGO III	T3	N1	MX/missing
161	FIGO III	T3	NX/missing	MX/missing
162	FIGO II	T2	N0	MX/missing
163	FIGO I	T1	N0	MX/missing
164	FIGO III	T3	N1	MX/missing
165	FIGO III	T3	NX/missing	MX/missing
166	FIGO II	T2	N0	MX/missing
167	FIGO III	T3	N1	MX/missing
168	FIGO II	T2	N0	MX/missing
169	FIGO III	T3	NX/missing	MX/missing
170	FIGO III	T3	N1	MX/missing
171	FIGO I	T1	N0	MX/missing
172	FIGO III	T3	N1	MX/missing
173	FIGO III	T3	N1	MX/missing
174	FIGO III	T3	NX/missing	MX/missing
175	FIGO IV	T3	NX/missing	M1
176	FIGO III	T3	N0	MX/missing
177	FIGO III	T3	N0	MX/missing
178	FIGO III	T3	N1	MX/missing
179	FIGO III	T3	N1	MX/missing
180	FIGO I	T1	N0	MX/missing
181	missing	TX/missing	NX/missing	MX/missing
182	FIGO III	T3	N0	MX/missing
183	FIGO III	T3	N1	MX/missing
184	FIGO III	T3	N0	MX/missing
185	FIGO I	T1	N0	MX/missing

S1 Table. Minimal data set.sav

	Age	Grouped_age	Median_age_HGSC	Death
149	59	50-59	not applicable	living
150	56	50-59	not applicable	living
151	80	80-89	not applicable	living
152	34	30-39	not applicable	living
153	79	70-79	> median age	living
154	38	30-39	< median age	dead
155	62	60-69	< median age	dead
156	63	60-69	> median age	living
157	77	70-79	> median age	dead
158	62	60-69	< median age	dead
159	63	60-69	> median age	dead
160	54	50-59	< median age	dead
161	65	60-69	> median age	dead
162	66	60-69	> median age	dead
163	76	70-79	> median age	dead
164	43	40-49	< median age	dead
165	80	80-89	> median age	dead
166	43	40-49	< median age	dead
167	50	50-59	< median age	dead
168	42	40-49	< median age	dead
169	69	60-69	> median age	dead
170	73	70-79	> median age	dead
171	59	50-59	< median age	dead
172	47	40-49	< median age	dead
173	53	50-59	not applicable	dead
174	85	80-89	> median age	living
175	73	70-79	not applicable	dead
176	52	50-59	< median age	dead
177	55	50-59	< median age	living
178	63	60-69	> median age	living
179	54	50-59	< median age	dead
180	62	60-69	< median age	living
181	83	80-89	> median age	dead
182	72	70-79	> median age	dead
183	50	50-59	< median age	dead
184	48	40-49	< median age	dead
185	58	50-59	< median age	living

S1 Table. Minimal data set.sav

	Survival_months	Survival_years	Predominant_staining_intensity
149	,20	,02	moderate staining
150	123,16	10,26	no staining
151	,00	,00	moderate staining
152	117,83	9,82	weak staining
153	127,79	10,65	moderate staining
154	6,05	,50	weak staining
155	22,39	1,87	moderate staining
156	126,35	10,53	weak staining
157	7,23	,60	moderate staining
158	18,81	1,57	moderate staining
159	113,92	9,49	strong staining
160	31,07	2,59	moderate staining
161	19,82	1,65	weak staining
162	40,90	3,41	moderate staining
163	119,08	9,92	moderate staining
164	26,53	2,21	moderate staining
165	12,69	1,06	weak staining
166	92,12	7,68	strong staining
167	42,97	3,58	moderate staining
168	36,92	3,08	moderate staining
169	33,04	2,75	strong staining
170	1,61	,13	strong staining
171	32,71	2,73	strong staining
172	101,72	8,48	moderate staining
173	1,74	,15	strong staining
174	,62	,05	moderate staining
175	10,95	,91	moderate staining
176	64,21	5,35	weak staining
177	37,55	3,13	moderate staining
178	2,33	,19	moderate staining
179	38,37	3,20	moderate staining
180	120,23	10,02	moderate staining
181	21,76	1,81	moderate staining
182	48,69	4,06	weak staining
183	35,11	2,93	moderate staining
184	51,39	4,28	strong staining
185	118,39	9,87	strong staining

S1 Table. Minimal data set.sav

	Percentage_of_positive_stained_cells	Immunoreactive_score
149	>80%	8
150	no staining	0
151	>80%	8
152	>80%	4
153	>80%	8
154	>80%	4
155	>80%	8
156	>80%	4
157	>80%	8
158	>80%	8
159	>80%	12
160	51-80%	6
161	51-80%	3
162	51-80%	6
163	>80%	8
164	>80%	8
165	51-80%	3
166	>80%	12
167	51-80%	6
168	>80%	8
169	>80%	12
170	>80%	12
171	>80%	12
172	>80%	8
173	>80%	12
174	>80%	8
175	51-80%	6
176	51-80%	3
177	51-80%	6
178	>80%	8
179	51-80%	6
180	>80%	8
181	>80%	8
182	>80%	4
183	>80%	8
184	>80%	12
185	51-80%	9

	Positive_IRS	Cutoff_IRS	Grouped_IRS
149	IRS >2	IRS ≤8	IRS 3-8
150	IRS ≤2	IRS ≤8	IRS 0-2
151	IRS >2	IRS ≤8	IRS 3-8
152	IRS >2	IRS ≤8	IRS 3-8
153	IRS >2	IRS ≤8	IRS 3-8
154	IRS >2	IRS ≤8	IRS 3-8
155	IRS >2	IRS ≤8	IRS 3-8
156	IRS >2	IRS ≤8	IRS 3-8
157	IRS >2	IRS ≤8	IRS 3-8
158	IRS >2	IRS ≤8	IRS 3-8
159	IRS >2	IRS >8	IRS 9-12
160	IRS >2	IRS ≤8	IRS 3-8
161	IRS >2	IRS ≤8	IRS 3-8
162	IRS >2	IRS ≤8	IRS 3-8
163	IRS >2	IRS ≤8	IRS 3-8
164	IRS >2	IRS ≤8	IRS 3-8
165	IRS >2	IRS ≤8	IRS 3-8
166	IRS >2	IRS >8	IRS 9-12
167	IRS >2	IRS ≤8	IRS 3-8
168	IRS >2	IRS ≤8	IRS 3-8
169	IRS >2	IRS >8	IRS 9-12
170	IRS >2	IRS >8	IRS 9-12
171	IRS >2	IRS >8	IRS 9-12
172	IRS >2	IRS ≤8	IRS 3-8
173	IRS >2	IRS >8	IRS 9-12
174	IRS >2	IRS ≤8	IRS 3-8
175	IRS >2	IRS ≤8	IRS 3-8
176	IRS >2	IRS ≤8	IRS 3-8
177	IRS >2	IRS ≤8	IRS 3-8
178	IRS >2	IRS ≤8	IRS 3-8
179	IRS >2	IRS ≤8	IRS 3-8
180	IRS >2	IRS ≤8	IRS 3-8
181	IRS >2	IRS ≤8	IRS 3-8
182	IRS >2	IRS ≤8	IRS 3-8
183	IRS >2	IRS ≤8	IRS 3-8
184	IRS >2	IRS >8	IRS 9-12
185	IRS >2	IRS >8	IRS 9-12

S1 Table. Minimal data set.sav

	Presence_of_strong_SI	Percentage_of_strong_SI
149	no areas of strong staining intensity	no strong staining intensity
150	no areas of strong staining intensity	no strong staining intensity
151	areas of strong staining intensity	10-50%
152	areas of strong staining intensity	<10%
153	areas of strong staining intensity	10-50%
154	no areas of strong staining intensity	no strong staining intensity
155	areas of strong staining intensity	<10%
156	no areas of strong staining intensity	no strong staining intensity
157	areas of strong staining intensity	<10%
158	areas of strong staining intensity	<10%
159	areas of strong staining intensity	51-80%
160	areas of strong staining intensity	<10%
161	areas of strong staining intensity	<10%
162	areas of strong staining intensity	<10%
163	areas of strong staining intensity	<10%
164	no areas of strong staining intensity	no strong staining intensity
165	no areas of strong staining intensity	no strong staining intensity
166	areas of strong staining intensity	51-80%
167	areas of strong staining intensity	10-50%
168	no areas of strong staining intensity	no strong staining intensity
169	areas of strong staining intensity	51-80%
170	areas of strong staining intensity	>80%
171	areas of strong staining intensity	10-50%
172	areas of strong staining intensity	<10%
173	areas of strong staining intensity	51-80%
174	areas of strong staining intensity	10-50%
175	areas of strong staining intensity	<10%
176	no areas of strong staining intensity	no strong staining intensity
177	areas of strong staining intensity	<10%
178	areas of strong staining intensity	<10%
179	areas of strong staining intensity	<10%
180	areas of strong staining intensity	10-50%
181	no areas of strong staining intensity	no strong staining intensity
182	no areas of strong staining intensity	no strong staining intensity
183	areas of strong staining intensity	10-50%
184	areas of strong staining intensity	51-80%
185	areas of strong staining intensity	10-50%

S1 Table. Minimal data set.sav

	Cohort	Histology	Grading
186	Ovarian carcinoma	serous high-grade	G3
187	Ovarian carcinoma	serous high-grade	G3
188	Ovarian carcinoma	serous high-grade	G3
189	Ovarian carcinoma	serous high-grade	G3
190	Ovarian carcinoma	serous high-grade	G3
191	Ovarian carcinoma	serous high-grade	G3
192	Ovarian carcinoma	serous high-grade	G3
193	Ovarian carcinoma	serous high-grade	G3
194	Ovarian carcinoma	serous high-grade	G3
195	Ovarian carcinoma	undifferentiated	G3
196	Ovarian carcinoma	serous high-grade	G3
197	Ovarian carcinoma	serous high-grade	G3
198	Ovarian carcinoma	serous high-grade	G3
199	Ovarian carcinoma	serous high-grade	G3
200	Ovarian carcinoma	serous high-grade	G3
201	Ovarian carcinoma	serous high-grade	G3
202	Ovarian carcinoma	serous high-grade	G3
203	Ovarian carcinoma	serous high-grade	G3
204	Ovarian carcinoma	serous high-grade	G3
205	Ovarian carcinoma	serous high-grade	G3
206	Ovarian carcinoma	serous high-grade	G3
207	Ovarian carcinoma	serous high-grade	G3
208	Ovarian carcinoma	serous high-grade	G3
209	Ovarian carcinoma	serous high-grade	G3
210	Ovarian carcinoma	serous high-grade	G3
211	Ovarian carcinoma	serous low-grade	G1
212	Ovarian carcinoma	serous low-grade	G1
213	Ovarian carcinoma	serous low-grade	G1
214	Ovarian carcinoma	serous low-grade	G1
215	Ovarian carcinoma	serous low-grade	G1
216	Ovarian carcinoma	serous low-grade	G1
217	Ovarian carcinoma	endometrioid	G2
218	Ovarian carcinoma	endometrioid	G2
219	Ovarian carcinoma	mucinous	G2
220	Ovarian carcinoma	serous high-grade	G3
221	Ovarian carcinoma	serous high-grade	G3
222	Ovarian carcinoma	serous high-grade	G3

S1 Table. Minimal data set.sav

	FIGO	Primary_tumor_expansion	Nodal_status	Distant_metastasis
186	FIGO III	T3	N1	MX/missing
187	FIGO III	T3	NX/missing	MX/missing
188	FIGO III	T3	NX/missing	MX/missing
189	FIGO I	T1	N0	MX/missing
190	FIGO III	T3	NX/missing	MX/missing
191	FIGO II	T2	N0	MX/missing
192	FIGO III	T3	NX/missing	MX/missing
193	FIGO III	T3	NX/missing	MX/missing
194	FIGO III	T3	NX/missing	MX/missing
195	FIGO III	T3	N1	MX/missing
196	FIGO III	T3	NX/missing	MX/missing
197	FIGO III	T3	NX/missing	MX/missing
198	FIGO II	T2	N0	MX/missing
199	FIGO III	T3	N0	MX/missing
200	FIGO II	T2	N0	MX/missing
201	FIGO III	T3	N0	M0
202	FIGO III	T3	N0	MX/missing
203	FIGO III	T3	N1	MX/missing
204	FIGO III	T1	N1	MX/missing
205	FIGO III	T3	N1	MX/missing
206	FIGO III	T3	N0	M0
207	FIGO III	T3	N0	MX/missing
208	FIGO I	T1	NX/missing	MX/missing
209	missing	TX/missing	NX/missing	MX/missing
210	FIGO III	T3	N0	M0
211	FIGO III	T3	N1	MX/missing
212	FIGO III	T3	N1	MX/missing
213	FIGO III	T3	NX/missing	MX/missing
214	FIGO III	T3	N1	MX/missing
215	FIGO III	TX/missing	N1	MX/missing
216	FIGO IV	TX/missing	NX/missing	M1
217	FIGO I	T1	N0	MX/missing
218	FIGO I	T1	N0	MX/missing
219	FIGO III	T3	NX/missing	MX/missing
220	FIGO III	T3	N0	MX/missing
221	FIGO III	T3	NX/missing	MX/missing
222	FIGO III	T3	NX/missing	MX/missing

S1 Table. Minimal data set.sav

	Age	Grouped_age	Median_age_HGSC	Death
186	73	70-79	> median age	living
187	76	70-79	> median age	dead
188	56	50-59	< median age	dead
189	50	50-59	< median age	living
190	67	60-69	> median age	dead
191	66	60-69	> median age	living
192	61	60-69	< median age	dead
193	78	70-79	> median age	dead
194	70	70-79	> median age	dead
195	67	60-69	not applicable	dead
196	62	60-69	< median age	dead
197	61	60-69	< median age	dead
198	76	70-79	> median age	living
199	74	70-79	> median age	dead
200	78	70-79	> median age	dead
201	46	40-49	< median age	living
202	54	50-59	< median age	living
203	66	60-69	> median age	dead
204	81	80-89	> median age	living
205	66	60-69	> median age	dead
206	61	60-69	< median age	dead
207	51	50-59	< median age	dead
208	55	50-59	< median age	dead
209	43	40-49	< median age	dead
210	67	60-69	> median age	dead
211	51	50-59	not applicable	dead
212	73	70-79	not applicable	dead
213	64	60-69	not applicable	dead
214	57	50-59	not applicable	dead
215	58	50-59	not applicable	dead
216	66	60-69	not applicable	living
217	79	70-79	not applicable	dead
218	75	70-79	not applicable	living
219	79	70-79	not applicable	dead
220	58	50-59	< median age	dead
221	58	50-59	< median age	dead
222	51	50-59	< median age	dead

S1 Table. Minimal data set.sav

	Survival_months	Survival_years	Predominant_staining_intensity
186	35,77	2,98	strong staining
187	16,80	1,40	strong staining
188	85,97	7,16	moderate staining
189	117,80	9,82	weak staining
190	24,00	2,00	weak staining
191	50,79	4,23	moderate staining
192	14,60	1,22	strong staining
193	1,25	,10	strong staining
194	14,40	1,20	moderate staining
195	49,02	4,08	moderate staining
196	14,86	1,24	weak staining
197	9,37	,78	strong staining
198	116,98	9,75	moderate staining
199	112,01	9,33	strong staining
200	50,76	4,23	strong staining
201	106,52	8,88	strong staining
202	55,27	4,61	weak staining
203	49,41	4,12	moderate staining
204	106,95	8,91	weak staining
205	100,01	8,33	weak staining
206	77,69	6,47	strong staining
207	35,28	2,94	weak staining
208	43,07	3,59	strong staining
209	3,85	,32	strong staining
210	35,44	2,95	weak staining
211	30,31	2,53	weak staining
212	10,85	,90	moderate staining
213	56,91	4,74	moderate staining
214	61,94	5,16	moderate staining
215	51,85	4,32	weak staining
216	35,77	2,98	weak staining
217	6,61	,55	moderate staining
218	38,63	3,22	moderate staining
219	8,52	,71	moderate staining
220	89,52	7,46	strong staining
221	22,82	1,90	strong staining
222	59,93	4,99	moderate staining

S1 Table. Minimal data set.sav

	Percentage_of_positive_stained_cells	Immunoreactive_score
186	>80%	12
187	>80%	12
188	>80%	8
189	>80%	4
190	51-80%	3
191	>80%	8
192	51-80%	9
193	>80%	12
194	>80%	8
195	>80%	8
196	51-80%	3
197	>80%	12
198	<10%	2
199	>80%	12
200	>80%	12
201	>80%	12
202	51-80%	3
203	>80%	8
204	51-80%	3
205	51-80%	3
206	>80%	12
207	10-50%	2
208	>80%	12
209	>80%	12
210	>80%	4
211	<10%	1
212	>80%	8
213	>80%	8
214	>80%	8
215	10-50%	2
216	>80%	4
217	>80%	8
218	51-80%	6
219	>80%	8
220	>80%	12
221	>80%	12
222	>80%	8

S1 Table. Minimal data set.sav

	Positive_IRS	Cutoff_IRS	Grouped_IRS
186	IRS >2	IRS >8	IRS 9-12
187	IRS >2	IRS >8	IRS 9-12
188	IRS >2	IRS ≤8	IRS 3-8
189	IRS >2	IRS ≤8	IRS 3-8
190	IRS >2	IRS ≤8	IRS 3-8
191	IRS >2	IRS ≤8	IRS 3-8
192	IRS >2	IRS >8	IRS 9-12
193	IRS >2	IRS >8	IRS 9-12
194	IRS >2	IRS ≤8	IRS 3-8
195	IRS >2	IRS ≤8	IRS 3-8
196	IRS >2	IRS ≤8	IRS 3-8
197	IRS >2	IRS >8	IRS 9-12
198	IRS ≤2	IRS ≤8	IRS 0-2
199	IRS >2	IRS >8	IRS 9-12
200	IRS >2	IRS >8	IRS 9-12
201	IRS >2	IRS >8	IRS 9-12
202	IRS >2	IRS ≤8	IRS 3-8
203	IRS >2	IRS ≤8	IRS 3-8
204	IRS >2	IRS ≤8	IRS 3-8
205	IRS >2	IRS ≤8	IRS 3-8
206	IRS >2	IRS >8	IRS 9-12
207	IRS ≤2	IRS ≤8	IRS 0-2
208	IRS >2	IRS >8	IRS 9-12
209	IRS >2	IRS >8	IRS 9-12
210	IRS >2	IRS ≤8	IRS 3-8
211	IRS ≤2	IRS ≤8	IRS 0-2
212	IRS >2	IRS ≤8	IRS 3-8
213	IRS >2	IRS ≤8	IRS 3-8
214	IRS >2	IRS ≤8	IRS 3-8
215	IRS ≤2	IRS ≤8	IRS 0-2
216	IRS >2	IRS ≤8	IRS 3-8
217	IRS >2	IRS ≤8	IRS 3-8
218	IRS >2	IRS ≤8	IRS 3-8
219	IRS >2	IRS ≤8	IRS 3-8
220	IRS >2	IRS >8	IRS 9-12
221	IRS >2	IRS >8	IRS 9-12
222	IRS >2	IRS ≤8	IRS 3-8

S1 Table. Minimal data set.sav

	Presence_of_strong_SI	Percentage_of_strong_SI
186	areas of strong staining intensity	51-80%
187	areas of strong staining intensity	51-80%
188	areas of strong staining intensity	<10%
189	areas of strong staining intensity	<10%
190	areas of strong staining intensity	<10%
191	areas of strong staining intensity	10-50%
192	areas of strong staining intensity	10-50%
193	areas of strong staining intensity	>80%
194	areas of strong staining intensity	10-50%
195	areas of strong staining intensity	10-50%
196	no areas of strong staining intensity	no strong staining intensity
197	areas of strong staining intensity	>80%
198	no areas of strong staining intensity	no strong staining intensity
199	areas of strong staining intensity	51-80%
200	areas of strong staining intensity	51-80%
201	areas of strong staining intensity	>80%
202	no areas of strong staining intensity	no strong staining intensity
203	areas of strong staining intensity	<10%
204	areas of strong staining intensity	<10%
205	areas of strong staining intensity	<10%
206	areas of strong staining intensity	>80%
207	no areas of strong staining intensity	no strong staining intensity
208	areas of strong staining intensity	10-50%
209	areas of strong staining intensity	51-80%
210	no areas of strong staining intensity	no strong staining intensity
211	no areas of strong staining intensity	no strong staining intensity
212	areas of strong staining intensity	<10%
213	areas of strong staining intensity	<10%
214	areas of strong staining intensity	10-50%
215	no areas of strong staining intensity	no strong staining intensity
216	no areas of strong staining intensity	no strong staining intensity
217	areas of strong staining intensity	<10%
218	no areas of strong staining intensity	no strong staining intensity
219	areas of strong staining intensity	10-50%
220	areas of strong staining intensity	51-80%
221	areas of strong staining intensity	51-80%
222	areas of strong staining intensity	<10%

	Cohort	Histology	Grading
223	Ovarian carcinoma	serous high-grade	G3
224	Ovarian carcinoma	serous high-grade	G3
225	Ovarian carcinoma	serous high-grade	G3
226	Ovarian carcinoma	serous high-grade	G3
227	Ovarian carcinoma	serous high-grade	G3
228	Ovarian carcinoma	serous high-grade	G3
229	Ovarian carcinoma	serous high-grade	G3
230	Ovarian carcinoma	serous high-grade	G3
231	Ovarian carcinoma	clear cell	G3
232	Ovarian carcinoma	serous high-grade	G3
233	Ovarian carcinoma	serous high-grade	G3
234	Ovarian carcinoma	serous high-grade	G3
235	Ovarian carcinoma	serous high-grade	G3
236	Ovarian carcinoma	serous high-grade	G3
237	Ovarian carcinoma	serous high-grade	G3
238	Ovarian carcinoma	serous high-grade	G3
239	Ovarian carcinoma	serous high-grade	G3
240	Ovarian carcinoma	serous high-grade	G3
241	Ovarian carcinoma	serous high-grade	G3
242	Ovarian carcinoma	serous high-grade	G3
243	Ovarian carcinoma	serous high-grade	G3
244	Ovarian carcinoma	serous high-grade	G3
245	Ovarian carcinoma	serous high-grade	G3
246	Ovarian carcinoma	serous high-grade	G3
247	Ovarian carcinoma	serous high-grade	G3
248	Ovarian carcinoma	endometrioid	G3
249	Ovarian carcinoma	serous high-grade	G3
250	Ovarian carcinoma	serous high-grade	G3
251	Ovarian carcinoma	serous high-grade	G3
252	Ovarian carcinoma	serous high-grade	G3
253	Ovarian carcinoma	clear cell	G3
254	Ovarian carcinoma	serous high-grade	G3
255	Ovarian carcinoma	serous high-grade	G3
256	Ovarian carcinoma	serous high-grade	G3
257	Ovarian carcinoma	serous high-grade	G3
258	Ovarian carcinoma	serous high-grade	G3
259	Ovarian carcinoma	serous high-grade	G3

S1 Table. Minimal data set.sav

	FIGO	Primary_tumor_expansion	Nodal_status	Distant_metastasis
223	FIGO III	T3	N0	MX/missing
224	FIGO III	T3	N1	MX/missing
225	FIGO III	T3	NX/missing	MX/missing
226	FIGO III	T3	NX/missing	MX/missing
227	FIGO III	T3	N1	MX/missing
228	FIGO IV	T3	N0	M1
229	FIGO III	T3	NX/missing	MX/missing
230	FIGO III	T3	NX/missing	MX/missing
231	FIGO III	T3	N1	MX/missing
232	FIGO IV	T3	NX/missing	M1
233	FIGO IV	T3	NX/missing	M1
234	FIGO III	T3	NX/missing	MX/missing
235	FIGO III	T3	N0	MX/missing
236	FIGO III	T3	NX/missing	MX/missing
237	FIGO III	T3	N1	MX/missing
238	FIGO III	T3	NX/missing	MX/missing
239	FIGO III	T3	N1	MX/missing
240	FIGO III	T3	N1	MX/missing
241	FIGO III	T3	N1	MX/missing
242	FIGO IV	T3	NX/missing	M1
243	FIGO IV	T3	NX/missing	M1
244	FIGO I	T1	N0	MX/missing
245	FIGO III	T3	NX/missing	MX/missing
246	FIGO III	T3	NX/missing	MX/missing
247	FIGO III	T3	N1	MX/missing
248	FIGO II	T2	N0	MX/missing
249	FIGO II	T2	N0	MX/missing
250	FIGO III	T3	NX/missing	MX/missing
251	FIGO III	T3	N0	MX/missing
252	FIGO III	T3	N1	MX/missing
253	FIGO I	T1	N0	MX/missing
254	FIGO III	T3	N1	MX/missing
255	FIGO III	T3	N1	MX/missing
256	FIGO III	T3	NX/missing	MX/missing
257	FIGO III	T3	NX/missing	MX/missing
258	FIGO III	T3	N0	MX/missing
259	FIGO III	T3	NX/missing	MX/missing

S1 Table. Minimal data set.sav

	Age	Grouped_age	Median_age_HGSC	Death
223	51	50-59	< median age	living
224	45	40-49	< median age	living
225	79	70-79	> median age	dead
226	66	60-69	> median age	dead
227	63	60-69	< median age	living
228	66	60-69	> median age	dead
229	48	40-49	< median age	dead
230	67	60-69	> median age	dead
231	49	40-49	not applicable	dead
232	55	50-59	< median age	dead
233	68	60-69	> median age	dead
234	41	40-49	< median age	dead
235	52	50-59	< median age	dead
236	61	60-69	< median age	dead
237	47	40-49	< median age	living
238	66	60-69	> median age	dead
239	56	50-59	< median age	dead
240	61	60-69	< median age	dead
241	67	60-69	> median age	dead
242	46	40-49	< median age	living
243	63	60-69	> median age	dead
244	60	60-69	< median age	living
245	53	50-59	< median age	living
246	69	60-69	> median age	dead
247	63	60-69	> median age	dead
248	67	60-69	not applicable	living
249	70	70-79	> median age	dead
250	63	60-69	> median age	living
251	61	60-69	< median age	dead
252	64	60-69	> median age	dead
253	62	60-69	not applicable	living
254	47	40-49	< median age	dead
255	52	50-59	< median age	living
256	41	40-49	< median age	dead
257	65	60-69	> median age	dead
258	66	60-69	> median age	dead
259	90	90-99	> median age	missing

S1 Table. Minimal data set.sav

	Survival_months	Survival_years	Predominant_staining_intensity
223	48,33	4,03	moderate staining
224	55,63	4,64	strong staining
225	55,59	4,63	strong staining
226	16,80	1,40	moderate staining
227	114,77	9,56	weak staining
228	65,16	5,43	moderate staining
229	36,99	3,08	moderate staining
230	86,66	7,22	strong staining
231	6,05	,50	moderate staining
232	27,95	2,33	moderate staining
233	9,44	,79	weak staining
234	40,01	3,33	strong staining
235	116,88	9,74	strong staining
236	5,72	,48	weak staining
237	112,41	9,37	weak staining
238	11,31	,94	moderate staining
239	68,25	5,69	weak staining
240	1,08	,09	strong staining
241	28,18	2,35	moderate staining
242	40,14	3,35	weak staining
243	71,57	5,96	strong staining
244	39,81	3,32	strong staining
245	,72	,06	strong staining
246	14,83	1,24	strong staining
247	11,84	,99	weak staining
248	39,19	3,27	moderate staining
249	81,80	6,82	weak staining
250	109,81	9,15	moderate staining
251	59,64	4,97	weak staining
252	56,81	4,73	strong staining
253	37,38	3,12	weak staining
254	9,67	,81	moderate staining
255	112,08	9,34	moderate staining
256	32,02	2,67	weak staining
257	24,59	2,05	weak staining
258	6,61	,55	strong staining
259	39,75	3,31	weak staining

	Percentage_of_positive_stained_cells	Immunoreactive_score
223	>80%	8
224	>80%	12
225	>80%	12
226	>80%	8
227	10-50%	2
228	>80%	8
229	>80%	8
230	>80%	12
231	>80%	8
232	>80%	8
233	>80%	4
234	>80%	12
235	>80%	12
236	<10%	1
237	>80%	4
238	>80%	8
239	10-50%	2
240	>80%	12
241	<10%	2
242	>80%	4
243	>80%	12
244	>80%	12
245	>80%	12
246	>80%	12
247	>80%	4
248	51-80%	6
249	10-50%	2
250	>80%	8
251	<10%	1
252	51-80%	9
253	>80%	4
254	>80%	8
255	>80%	8
256	>80%	4
257	>80%	4
258	>80%	12
259	<10%	1

S1 Table. Minimal data set.sav

	Positive_IRS	Cutoff_IRS	Grouped_IRS
223	IRS >2	IRS ≤8	IRS 3-8
224	IRS >2	IRS >8	IRS 9-12
225	IRS >2	IRS >8	IRS 9-12
226	IRS >2	IRS ≤8	IRS 3-8
227	IRS ≤2	IRS ≤8	IRS 0-2
228	IRS >2	IRS ≤8	IRS 3-8
229	IRS >2	IRS ≤8	IRS 3-8
230	IRS >2	IRS >8	IRS 9-12
231	IRS >2	IRS ≤8	IRS 3-8
232	IRS >2	IRS ≤8	IRS 3-8
233	IRS >2	IRS ≤8	IRS 3-8
234	IRS >2	IRS >8	IRS 9-12
235	IRS >2	IRS >8	IRS 9-12
236	IRS ≤2	IRS ≤8	IRS 0-2
237	IRS >2	IRS ≤8	IRS 3-8
238	IRS >2	IRS ≤8	IRS 3-8
239	IRS ≤2	IRS ≤8	IRS 0-2
240	IRS >2	IRS >8	IRS 9-12
241	IRS ≤2	IRS ≤8	IRS 0-2
242	IRS >2	IRS ≤8	IRS 3-8
243	IRS >2	IRS >8	IRS 9-12
244	IRS >2	IRS >8	IRS 9-12
245	IRS >2	IRS >8	IRS 9-12
246	IRS >2	IRS >8	IRS 9-12
247	IRS >2	IRS ≤8	IRS 3-8
248	IRS >2	IRS ≤8	IRS 3-8
249	IRS ≤2	IRS ≤8	IRS 0-2
250	IRS >2	IRS ≤8	IRS 3-8
251	IRS ≤2	IRS ≤8	IRS 0-2
252	IRS >2	IRS >8	IRS 9-12
253	IRS >2	IRS ≤8	IRS 3-8
254	IRS >2	IRS ≤8	IRS 3-8
255	IRS >2	IRS ≤8	IRS 3-8
256	IRS >2	IRS ≤8	IRS 3-8
257	IRS >2	IRS ≤8	IRS 3-8
258	IRS >2	IRS >8	IRS 9-12
259	IRS ≤2	IRS ≤8	IRS 0-2

	Presence_of_strong_SI	Percentage_of_strong_SI
223	areas of strong staining intensity	<10%
224	areas of strong staining intensity	10-50%
225	areas of strong staining intensity	51-80%
226	no areas of strong staining intensity	no strong staining intensity
227	no areas of strong staining intensity	no strong staining intensity
228	no areas of strong staining intensity	no strong staining intensity
229	areas of strong staining intensity	10-50%
230	areas of strong staining intensity	>80%
231	areas of strong staining intensity	10-50%
232	areas of strong staining intensity	<10%
233	areas of strong staining intensity	<10%
234	areas of strong staining intensity	10-50%
235	areas of strong staining intensity	10-50%
236	no areas of strong staining intensity	no strong staining intensity
237	no areas of strong staining intensity	no strong staining intensity
238	areas of strong staining intensity	<10%
239	no areas of strong staining intensity	no strong staining intensity
240	areas of strong staining intensity	>80%
241	no areas of strong staining intensity	no strong staining intensity
242	no areas of strong staining intensity	no strong staining intensity
243	areas of strong staining intensity	10-50%
244	areas of strong staining intensity	10-50%
245	areas of strong staining intensity	10-50%
246	areas of strong staining intensity	10-50%
247	no areas of strong staining intensity	no strong staining intensity
248	areas of strong staining intensity	<10%
249	no areas of strong staining intensity	no strong staining intensity
250	areas of strong staining intensity	10-50%
251	no areas of strong staining intensity	no strong staining intensity
252	areas of strong staining intensity	10-50%
253	areas of strong staining intensity	<10%
254	areas of strong staining intensity	10-50%
255	areas of strong staining intensity	<10%
256	no areas of strong staining intensity	no strong staining intensity
257	no areas of strong staining intensity	no strong staining intensity
258	areas of strong staining intensity	10-50%
259	no areas of strong staining intensity	no strong staining intensity

S1 Table. Minimal data set.sav

	Cohort	Histology	Grading
260	Ovarian carcinoma	serous high-grade	G3
261	Ovarian carcinoma	serous high-grade	G3
262	Ovarian carcinoma	serous high-grade	G3
263	Ovarian carcinoma	serous high-grade	G3
264	Ovarian carcinoma	serous high-grade	G3
265	Ovarian carcinoma	serous high-grade	G3
266	Ovarian carcinoma	undifferentiated	G3
267	Ovarian carcinoma	serous high-grade	G3
268	Ovarian carcinoma	serous high-grade	G3
269	Ovarian carcinoma	serous high-grade	G3
270	Ovarian carcinoma	serous high-grade	G3
271	Ovarian carcinoma	serous high-grade	G3
272	Ovarian carcinoma	serous high-grade	G3
273	Ovarian carcinoma	serous high-grade	G3
274	Ovarian carcinoma	endometrioid	G1
275	Ovarian carcinoma	serous low-grade	G1
276	Ovarian carcinoma	serous low-grade	G1
277	Ovarian carcinoma	serous low-grade	G1
278	Ovarian carcinoma	endometrioid	G1
279	Ovarian carcinoma	serous low-grade	G1
280	Ovarian carcinoma	serous low-grade	G1
281	Ovarian carcinoma	endometrioid	G2
282	Ovarian carcinoma	mucinous	G2
283	Ovarian carcinoma	serous high-grade	G3
284	Ovarian carcinoma	serous high-grade	G3
285	Ovarian carcinoma	serous high-grade	G3
286	Ovarian carcinoma	serous high-grade	G3
287	Ovarian carcinoma	serous high-grade	G3
288	Ovarian carcinoma	serous high-grade	G3
289	Ovarian carcinoma	serous high-grade	G3
290	Ovarian carcinoma	serous high-grade	G3
291	Ovarian carcinoma	serous high-grade	G3
292	Ovarian carcinoma	serous high-grade	G3
293	Ovarian carcinoma	serous high-grade	G3
294	Ovarian carcinoma	serous high-grade	G3
295	Ovarian carcinoma	serous high-grade	G3
296	Ovarian carcinoma	endometrioid	G3

S1 Table. Minimal data set.sav

	FIGO	Primary_tumor_expansion	Nodal_status	Distant_metastasis
260	FIGO III	T3	N1	MX/missing
261	FIGO III	T3	NX/missing	MX/missing
262	FIGO IV	T3	N0	M1
263	FIGO II	T2	N0	M0
264	FIGO III	T3	NX/missing	MX/missing
265	FIGO III	T3	N1	MX/missing
266	FIGO III	T3	N1	MX/missing
267	FIGO III	T3	N1	MX/missing
268	FIGO IV	T3	NX/missing	M1
269	FIGO IV	T3	N1	M1
270	FIGO III	T3	N1	MX/missing
271	FIGO III	T3	N1	MX/missing
272	FIGO III	T3	N1	MX/missing
273	FIGO IV	T3	NX/missing	M1
274	FIGO I	T1	NX/missing	MX/missing
275	FIGO III	T3	N1	MX/missing
276	FIGO III	T3	N0	MX/missing
277	FIGO III	T3	NX/missing	MX/missing
278	FIGO I	T1	NX/missing	MX/missing
279	FIGO III	T3	N1	MX/missing
280	FIGO III	T3	N1	MX/missing
281	FIGO III	T2	N1	MX/missing
282	FIGO II	T2	NX/missing	MX/missing
283	FIGO III	T3	NX/missing	MX/missing
284	FIGO III	T3	N1	MX/missing
285	FIGO IV	T3	NX/missing	M1
286	FIGO III	T3	NX/missing	MX/missing
287	FIGO III	T3	N1	MX/missing
288	FIGO III	T3	N1	MX/missing
289	FIGO III	T3	NX/missing	MX/missing
290	FIGO III	T3	N1	MX/missing
291	FIGO III	T3	NX/missing	MX/missing
292	FIGO III	T3	NX/missing	MX/missing
293	FIGO III	T3	N0	MX/missing
294	FIGO III	T3	NX/missing	MX/missing
295	FIGO III	T3	N1	MX/missing
296	FIGO I	T1	NX/missing	MX/missing

S1 Table. Minimal data set.sav

	Age	Grouped_age	Median_age_HGSC	Death
260	44	40-49	< median age	dead
261	64	60-69	> median age	living
262	62	60-69	< median age	dead
263	38	30-39	< median age	dead
264	64	60-69	> median age	dead
265	70	70-79	> median age	living
266	42	40-49	not applicable	living
267	67	60-69	> median age	dead
268	55	50-59	< median age	dead
269	46	40-49	< median age	living
270	48	40-49	< median age	dead
271	43	40-49	< median age	living
272	44	40-49	< median age	dead
273	79	70-79	> median age	dead
274	82	80-89	not applicable	living
275	70	70-79	not applicable	dead
276	69	60-69	not applicable	dead
277	59	50-59	not applicable	living
278	23	20-29	not applicable	living
279	49	40-49	not applicable	dead
280	54	50-59	not applicable	living
281	51	50-59	not applicable	living
282	56	50-59	not applicable	living
283	57	50-59	< median age	dead
284	57	50-59	< median age	living
285	60	60-69	< median age	dead
286	68	60-69	> median age	dead
287	43	40-49	< median age	dead
288	48	40-49	< median age	dead
289	61	60-69	< median age	dead
290	63	60-69	< median age	dead
291	57	50-59	< median age	living
292	48	40-49	< median age	living
293	55	50-59	< median age	dead
294	74	70-79	> median age	dead
295	57	50-59	< median age	dead
296	32	30-39	not applicable	dead

S1 Table. Minimal data set.sav

	Survival_months	Survival_years	Predominant_staining_intensity
260	14,63	1,22	strong staining
261	104,25	8,69	weak staining
262	46,98	3,92	weak staining
263	100,83	8,40	weak staining
264	15,02	1,25	weak staining
265	,43	,04	moderate staining
266	1,38	,12	weak staining
267	27,06	2,25	weak staining
268	42,71	3,56	weak staining
269	31,43	2,62	moderate staining
270	19,20	1,60	moderate staining
271	6,90	,58	moderate staining
272	33,07	2,76	moderate staining
273	35,31	2,94	moderate staining
274	17,23	1,44	weak staining
275	25,74	2,15	moderate staining
276	59,41	4,95	moderate staining
277	96,43	8,04	weak staining
278	45,50	3,79	weak staining
279	45,93	3,83	weak staining
280	91,36	7,61	moderate staining
281	19,13	1,59	weak staining
282	44,22	3,68	moderate staining
283	60,03	5,00	moderate staining
284	1,84	,15	weak staining
285	38,20	3,18	weak staining
286	73,64	6,14	moderate staining
287	15,22	1,27	strong staining
288	28,87	2,41	moderate staining
289	93,40	7,78	moderate staining
290	31,40	2,62	strong staining
291	98,43	8,20	strong staining
292	missing	missing	weak staining
293	71,84	5,99	weak staining
294	25,28	2,11	weak staining
295	45,17	3,76	moderate staining
296	48,79	4,07	strong staining

S1 Table. Minimal data set.sav

	Percentage_of_positive_stained_cells	Immunoreactive_score
260	>80%	12
261	10-50%	2
262	>80%	4
263	>80%	4
264	51-80%	3
265	>80%	8
266	51-80%	3
267	>80%	4
268	>80%	4
269	>80%	8
270	51-80%	6
271	>80%	8
272	10-50%	4
273	>80%	8
274	>80%	4
275	>80%	8
276	>80%	8
277	>80%	4
278	10-50%	2
279	51-80%	3
280	>80%	8
281	>80%	4
282	>80%	8
283	>80%	8
284	51-80%	3
285	>80%	4
286	>80%	8
287	>80%	12
288	10-50%	4
289	>80%	8
290	>80%	12
291	>80%	12
292	>80%	4
293	>80%	4
294	>80%	4
295	>80%	8
296	>80%	12

	Positive_IRS	Cutoff_IRS	Grouped_IRS
260	IRS >2	IRS >8	IRS 9-12
261	IRS ≤2	IRS ≤8	IRS 0-2
262	IRS >2	IRS ≤8	IRS 3-8
263	IRS >2	IRS ≤8	IRS 3-8
264	IRS >2	IRS ≤8	IRS 3-8
265	IRS >2	IRS ≤8	IRS 3-8
266	IRS >2	IRS ≤8	IRS 3-8
267	IRS >2	IRS ≤8	IRS 3-8
268	IRS >2	IRS ≤8	IRS 3-8
269	IRS >2	IRS ≤8	IRS 3-8
270	IRS >2	IRS ≤8	IRS 3-8
271	IRS >2	IRS ≤8	IRS 3-8
272	IRS >2	IRS ≤8	IRS 3-8
273	IRS >2	IRS ≤8	IRS 3-8
274	IRS >2	IRS ≤8	IRS 3-8
275	IRS >2	IRS ≤8	IRS 3-8
276	IRS >2	IRS ≤8	IRS 3-8
277	IRS >2	IRS ≤8	IRS 3-8
278	IRS ≤2	IRS ≤8	IRS 0-2
279	IRS >2	IRS ≤8	IRS 3-8
280	IRS >2	IRS ≤8	IRS 3-8
281	IRS >2	IRS ≤8	IRS 3-8
282	IRS >2	IRS ≤8	IRS 3-8
283	IRS >2	IRS ≤8	IRS 3-8
284	IRS >2	IRS ≤8	IRS 3-8
285	IRS >2	IRS ≤8	IRS 3-8
286	IRS >2	IRS ≤8	IRS 3-8
287	IRS >2	IRS >8	IRS 9-12
288	IRS >2	IRS ≤8	IRS 3-8
289	IRS >2	IRS ≤8	IRS 3-8
290	IRS >2	IRS >8	IRS 9-12
291	IRS >2	IRS >8	IRS 9-12
292	IRS >2	IRS ≤8	IRS 3-8
293	IRS >2	IRS ≤8	IRS 3-8
294	IRS >2	IRS ≤8	IRS 3-8
295	IRS >2	IRS ≤8	IRS 3-8
296	IRS >2	IRS >8	IRS 9-12

S1 Table. Minimal data set.sav

	Presence_of_strong_SI	Percentage_of_strong_SI
260	areas of strong staining intensity	10-50%
261	no areas of strong staining intensity	no strong staining intensity
262	no areas of strong staining intensity	no strong staining intensity
263	no areas of strong staining intensity	no strong staining intensity
264	no areas of strong staining intensity	no strong staining intensity
265	areas of strong staining intensity	<10%
266	areas of strong staining intensity	<10%
267	no areas of strong staining intensity	no strong staining intensity
268	areas of strong staining intensity	<10%
269	no areas of strong staining intensity	no strong staining intensity
270	areas of strong staining intensity	<10%
271	no areas of strong staining intensity	no strong staining intensity
272	areas of strong staining intensity	<10%
273	areas of strong staining intensity	<10%
274	areas of strong staining intensity	<10%
275	areas of strong staining intensity	<10%
276	areas of strong staining intensity	10-50%
277	no areas of strong staining intensity	no strong staining intensity
278	no areas of strong staining intensity	no strong staining intensity
279	no areas of strong staining intensity	no strong staining intensity
280	areas of strong staining intensity	<10%
281	no areas of strong staining intensity	no strong staining intensity
282	areas of strong staining intensity	10-50%
283	areas of strong staining intensity	<10%
284	no areas of strong staining intensity	no strong staining intensity
285	no areas of strong staining intensity	no strong staining intensity
286	areas of strong staining intensity	<10%
287	areas of strong staining intensity	51-80%
288	areas of strong staining intensity	<10%
289	areas of strong staining intensity	<10%
290	areas of strong staining intensity	51-80%
291	areas of strong staining intensity	>80%
292	areas of strong staining intensity	<10%
293	areas of strong staining intensity	<10%
294	no areas of strong staining intensity	no strong staining intensity
295	areas of strong staining intensity	<10%
296	areas of strong staining intensity	51-80%

S1 Table. Minimal data set.sav

	Cohort	Histology	Grading
297	Ovarian carcinoma	serous high-grade	G3
298	Ovarian carcinoma	serous high-grade	G3
299	Ovarian carcinoma	serous high-grade	G3
300	Ovarian carcinoma	serous high-grade	G3
301	Ovarian carcinoma	serous high-grade	G3
302	Ovarian carcinoma	serous high-grade	G3
303	Ovarian carcinoma	serous high-grade	G3
304	Ovarian carcinoma	serous high-grade	G3
305	Ovarian carcinoma	serous high-grade	G3
306	Ovarian carcinoma	serous high-grade	G3
307	Ovarian carcinoma	serous high-grade	G3
308	Ovarian carcinoma	serous high-grade	G3
309	Ovarian carcinoma	serous high-grade	G3
310	Ovarian carcinoma	serous high-grade	G3
311	Ovarian carcinoma	serous high-grade	G3
312	Ovarian carcinoma	serous high-grade	G3
313	Ovarian carcinoma	serous high-grade	G3
314	Ovarian carcinoma	serous high-grade	G3
315	Ovarian carcinoma	serous high-grade	G3
316	Ovarian carcinoma	serous high-grade	G3
317	Ovarian carcinoma	serous high-grade	G3
318	Ovarian carcinoma	serous high-grade	G3
319	Ovarian carcinoma	serous high-grade	G3
320	Ovarian carcinoma	serous high-grade	G3
321	Ovarian carcinoma	serous high-grade	G3
322	Ovarian carcinoma	serous high-grade	G3
323	Ovarian carcinoma	serous high-grade	G3
324	Ovarian carcinoma	serous high-grade	G3
325	Ovarian carcinoma	serous high-grade	G3
326	Ovarian carcinoma	serous high-grade	G3
327	Ovarian carcinoma	serous high-grade	G3
328	Ovarian carcinoma	serous high-grade	G3
329	Ovarian carcinoma	serous high-grade	G3
330	Ovarian carcinoma	serous low-grade	G1
331	Ovarian carcinoma	serous low-grade	G1
332	Ovarian carcinoma	endometrioid	G1
333	Ovarian carcinoma	serous low-grade	G1

S1 Table. Minimal data set.sav

	FIGO	Primary_tumor_expansion	Nodal_status	Distant_metastasis
297	FIGO III	T3	NX/missing	MX/missing
298	FIGO III	T3	NX/missing	MX/missing
299	FIGO III	T3	N0	MX/missing
300	FIGO III	T3	N0	MX/missing
301	FIGO III	T3	NX/missing	MX/missing
302	FIGO III	T3	N0	MX/missing
303	FIGO I	T1	N0	MX/missing
304	FIGO III	T3	NX/missing	MX/missing
305	FIGO III	T3	NX/missing	MX/missing
306	FIGO IV	T3	N1	M1
307	FIGO IV	T3	NX/missing	M1
308	FIGO III	T3	NX/missing	MX/missing
309	FIGO III	T3	NX/missing	MX/missing
310	FIGO III	T3	N0	MX/missing
311	FIGO III	T3	NX/missing	MX/missing
312	FIGO IV	T3	NX/missing	M1
313	FIGO I	T1	NX/missing	MX/missing
314	FIGO IV	T3	NX/missing	M1
315	FIGO III	T3	N1	MX/missing
316	FIGO III	T2	N1	MX/missing
317	FIGO III	T3	NX/missing	MX/missing
318	FIGO III	T3	N1	MX/missing
319	FIGO III	T3	N0	MX/missing
320	FIGO III	T3	N1	MX/missing
321	FIGO III	T3	N1	MX/missing
322	FIGO III	T3	N0	MX/missing
323	FIGO III	T3	NX/missing	MX/missing
324	FIGO I	T1	NX/missing	MX/missing
325	FIGO III	T3	N0	MX/missing
326	FIGO III	T3	NX/missing	MX/missing
327	FIGO III	T3	N1	MX/missing
328	FIGO III	T3	N1	MX/missing
329	FIGO III	T3	NX/missing	MX/missing
330	FIGO III	T3	N1	M0
331	FIGO I	T1	N0	MX/missing
332	FIGO I	T1	N0	MX/missing
333	FIGO II	T2	N0	MX/missing

	Age	Grouped_age	Median_age_HGSC	Death
297	75	70-79	> median age	dead
298	62	60-69	< median age	dead
299	63	60-69	> median age	living
300	45	40-49	< median age	living
301	67	60-69	> median age	dead
302	64	60-69	> median age	living
303	56	50-59	< median age	living
304	60	60-69	< median age	dead
305	66	60-69	> median age	living
306	44	40-49	< median age	dead
307	75	70-79	> median age	dead
308	75	70-79	> median age	dead
309	66	60-69	> median age	dead
310	65	60-69	> median age	living
311	73	70-79	> median age	dead
312	78	70-79	> median age	dead
313	48	40-49	< median age	living
314	64	60-69	> median age	dead
315	81	80-89	> median age	dead
316	56	50-59	< median age	dead
317	76	70-79	> median age	dead
318	80	80-89	> median age	dead
319	64	60-69	> median age	living
320	62	60-69	< median age	dead
321	73	70-79	> median age	living
322	67	60-69	> median age	dead
323	66	60-69	> median age	dead
324	84	80-89	> median age	dead
325	52	50-59	< median age	dead
326	68	60-69	> median age	dead
327	57	50-59	< median age	dead
328	79	70-79	> median age	dead
329	53	50-59	< median age	living
330	35	30-39	not applicable	dead
331	71	70-79	not applicable	living
332	46	40-49	not applicable	living
333	61	60-69	not applicable	living

S1 Table. Minimal data set.sav

	Survival_months	Survival_years	Predominant_staining_intensity
297	27,22	2,27	strong staining
298	61,84	5,15	moderate staining
299	28,80	2,40	weak staining
300	110,89	9,24	weak staining
301	59,87	4,99	moderate staining
302	100,11	8,34	weak staining
303	99,68	8,31	strong staining
304	13,25	1,10	moderate staining
305	44,98	3,75	weak staining
306	8,28	,69	moderate staining
307	42,48	3,54	weak staining
308	31,76	2,65	weak staining
309	35,44	2,95	moderate staining
310	26,27	2,19	moderate staining
311	40,80	3,40	weak staining
312	29,26	2,44	weak staining
313	53,06	4,42	moderate staining
314	9,44	,79	moderate staining
315	,20	,02	weak staining
316	99,58	8,30	weak staining
317	23,24	1,94	moderate staining
318	14,76	1,23	weak staining
319	23,21	1,93	weak staining
320	46,62	3,88	weak staining
321	95,87	7,99	moderate staining
322	19,43	1,62	moderate staining
323	14,60	1,22	weak staining
324	20,12	1,68	weak staining
325	63,45	5,29	moderate staining
326	14,70	1,22	weak staining
327	54,87	4,57	weak staining
328	7,13	,59	moderate staining
329	21,04	1,75	moderate staining
330	37,22	3,10	moderate staining
331	88,80	7,40	moderate staining
332	16,21	1,35	moderate staining
333	15,25	1,27	weak staining

S1 Table. Minimal data set.sav

	Percentage_of_positive_stained_cells	Immunoreactive_score
297	51-80%	9
298	>80%	8
299	>80%	4
300	>80%	4
301	>80%	8
302	>80%	4
303	>80%	12
304	>80%	8
305	>80%	4
306	51-80%	6
307	51-80%	3
308	51-80%	3
309	>80%	8
310	>80%	8
311	51-80%	3
312	10-50%	2
313	>80%	8
314	51-80%	6
315	>80%	4
316	>80%	4
317	10-50%	4
318	>80%	4
319	>80%	4
320	51-80%	3
321	>80%	8
322	>80%	8
323	>80%	4
324	>80%	4
325	>80%	8
326	10-50%	2
327	>80%	4
328	51-80%	6
329	>80%	8
330	>80%	8
331	>80%	8
332	51-80%	6
333	51-80%	3

	Positive_IRS	Cutoff_IRS	Grouped_IRS
297	IRS >2	IRS >8	IRS 9-12
298	IRS >2	IRS ≤8	IRS 3-8
299	IRS >2	IRS ≤8	IRS 3-8
300	IRS >2	IRS ≤8	IRS 3-8
301	IRS >2	IRS ≤8	IRS 3-8
302	IRS >2	IRS ≤8	IRS 3-8
303	IRS >2	IRS >8	IRS 9-12
304	IRS >2	IRS ≤8	IRS 3-8
305	IRS >2	IRS ≤8	IRS 3-8
306	IRS >2	IRS ≤8	IRS 3-8
307	IRS >2	IRS ≤8	IRS 3-8
308	IRS >2	IRS ≤8	IRS 3-8
309	IRS >2	IRS ≤8	IRS 3-8
310	IRS >2	IRS ≤8	IRS 3-8
311	IRS >2	IRS ≤8	IRS 3-8
312	IRS ≤2	IRS ≤8	IRS 0-2
313	IRS >2	IRS ≤8	IRS 3-8
314	IRS >2	IRS ≤8	IRS 3-8
315	IRS >2	IRS ≤8	IRS 3-8
316	IRS >2	IRS ≤8	IRS 3-8
317	IRS >2	IRS ≤8	IRS 3-8
318	IRS >2	IRS ≤8	IRS 3-8
319	IRS >2	IRS ≤8	IRS 3-8
320	IRS >2	IRS ≤8	IRS 3-8
321	IRS >2	IRS ≤8	IRS 3-8
322	IRS >2	IRS ≤8	IRS 3-8
323	IRS >2	IRS ≤8	IRS 3-8
324	IRS >2	IRS ≤8	IRS 3-8
325	IRS >2	IRS ≤8	IRS 3-8
326	IRS ≤2	IRS ≤8	IRS 0-2
327	IRS >2	IRS ≤8	IRS 3-8
328	IRS >2	IRS ≤8	IRS 3-8
329	IRS >2	IRS ≤8	IRS 3-8
330	IRS >2	IRS ≤8	IRS 3-8
331	IRS >2	IRS ≤8	IRS 3-8
332	IRS >2	IRS ≤8	IRS 3-8
333	IRS >2	IRS ≤8	IRS 3-8

S1 Table. Minimal data set.sav

	Presence_of_strong_SI	Percentage_of_strong_SI
297	areas of strong staining intensity	10-50%
298	areas of strong staining intensity	<10%
299	no areas of strong staining intensity	no strong staining intensity
300	areas of strong staining intensity	<10%
301	areas of strong staining intensity	<10%
302	no areas of strong staining intensity	no strong staining intensity
303	areas of strong staining intensity	51-80%
304	areas of strong staining intensity	<10%
305	areas of strong staining intensity	<10%
306	areas of strong staining intensity	<10%
307	no areas of strong staining intensity	no strong staining intensity
308	no areas of strong staining intensity	no strong staining intensity
309	areas of strong staining intensity	<10%
310	areas of strong staining intensity	<10%
311	no areas of strong staining intensity	no strong staining intensity
312	no areas of strong staining intensity	no strong staining intensity
313	areas of strong staining intensity	<10%
314	areas of strong staining intensity	<10%
315	no areas of strong staining intensity	no strong staining intensity
316	no areas of strong staining intensity	no strong staining intensity
317	no areas of strong staining intensity	no strong staining intensity
318	no areas of strong staining intensity	no strong staining intensity
319	no areas of strong staining intensity	no strong staining intensity
320	areas of strong staining intensity	<10%
321	areas of strong staining intensity	<10%
322	areas of strong staining intensity	10-50%
323	no areas of strong staining intensity	no strong staining intensity
324	no areas of strong staining intensity	no strong staining intensity
325	areas of strong staining intensity	<10%
326	no areas of strong staining intensity	no strong staining intensity
327	no areas of strong staining intensity	no strong staining intensity
328	areas of strong staining intensity	<10%
329	areas of strong staining intensity	<10%
330	areas of strong staining intensity	<10%
331	areas of strong staining intensity	<10%
332	areas of strong staining intensity	<10%
333	no areas of strong staining intensity	no strong staining intensity

S1 Table. Minimal data set.sav

	Cohort	Histology	Grading
334	Ovarian carcinoma	serous low-grade	G1
335	Ovarian carcinoma	serous low-grade	G1
336	Ovarian carcinoma	mucinous	G1
337	Ovarian carcinoma	mucinous	G2
338	Ovarian carcinoma	endometrioid	G2
339	Ovarian carcinoma	endometrioid	G2
340	Ovarian carcinoma	endometrioid	G2
341	Ovarian carcinoma	seromucinous	G2
342	Ovarian carcinoma	endometrioid	G2
343	Ovarian carcinoma	mucinous	G2
344	Ovarian carcinoma	undifferentiated	G3
345	Ovarian carcinoma	serous high-grade	G3
346	Ovarian carcinoma	serous high-grade	G3
347	Ovarian carcinoma	serous high-grade	G3
348	Ovarian carcinoma	serous high-grade	G3
349	Ovarian carcinoma	serous high-grade	G3
350	Ovarian carcinoma	serous high-grade	G3
351	Ovarian carcinoma	serous high-grade	G3
352	Ovarian carcinoma	serous high-grade	G3
353	Ovarian carcinoma	serous high-grade	G3
354	Ovarian carcinoma	serous high-grade	G3
355	Ovarian carcinoma	serous high-grade	G3
356	Ovarian carcinoma	serous high-grade	G3
357	Ovarian carcinoma	serous high-grade	G3
358	Ovarian carcinoma	serous high-grade	G3
359	Ovarian carcinoma	undifferentiated	G3
360	Ovarian carcinoma	serous high-grade	G3
361	Ovarian carcinoma	undifferentiated	G3
362	Ovarian carcinoma	serous high-grade	G3
363	Ovarian carcinoma	serous high-grade	G3
364	Ovarian carcinoma	serous high-grade	G3
365	Ovarian carcinoma	serous high-grade	G3
366	Ovarian carcinoma	serous high-grade	G3
367	Ovarian carcinoma	serous high-grade	G3
368	Ovarian carcinoma	serous high-grade	G3
369	Ovarian carcinoma	undifferentiated	G3
370	Ovarian carcinoma	serous high-grade	G3

S1 Table. Minimal data set.sav

	FIGO	Primary_tumor_expansion	Nodal_status	Distant_metastasis
334	FIGO III	T3	N1	MX/missing
335	FIGO III	T3	N1	M0
336	FIGO IV	T3	NX/missing	M1
337	FIGO IV	T3	NX/missing	M1
338	FIGO III	T3	NX/missing	MX/missing
339	FIGO IV	T1	NX/missing	M1
340	FIGO II	T2	N0	MX/missing
341	FIGO I	T1	N0	MX/missing
342	FIGO I	T1	N0	MX/missing
343	FIGO III	T3	NX/missing	MX/missing
344	FIGO IV	T3	N1	M1
345	FIGO III	T3	N0	MX/missing
346	FIGO III	T3	NX/missing	MX/missing
347	FIGO III	T3	N1	MX/missing
348	FIGO III	T3	N1	MX/missing
349	FIGO III	T3	N1	MX/missing
350	FIGO IV	T3	N0	M1
351	FIGO III	T3	N0	MX/missing
352	FIGO IV	T3	N0	M1
353	FIGO IV	T3	N0	M1
354	FIGO IV	T3	NX/missing	M1
355	FIGO IV	T3	NX/missing	M1
356	FIGO III	T3	N0	MX/missing
357	FIGO III	T3	N0	MX/missing
358	FIGO III	T3	NX/missing	MX/missing
359	FIGO III	T3	NX/missing	MX/missing
360	FIGO III	T3	N1	MX/missing
361	FIGO III	T3	N1	MX/missing
362	FIGO III	T1	N1	MX/missing
363	FIGO III	T3	N1	MX/missing
364	FIGO II	T2	NX/missing	MX/missing
365	FIGO II	T2	NX/missing	MX/missing
366	FIGO III	T3	N0	MX/missing
367	FIGO III	T3	N1	MX/missing
368	FIGO IV	T2	NX/missing	M1
369	FIGO III	T3	NX/missing	MX/missing
370	FIGO III	T3	NX/missing	MX/missing

S1 Table. Minimal data set.sav

	Age	Grouped_age	Median_age_HGSC	Death
334	46	40-49	not applicable	living
335	50	50-59	not applicable	dead
336	76	70-79	not applicable	dead
337	69	60-69	not applicable	dead
338	54	50-59	not applicable	dead
339	65	60-69	not applicable	dead
340	48	40-49	not applicable	living
341	32	30-39	not applicable	living
342	65	60-69	not applicable	living
343	44	40-49	not applicable	living
344	87	80-89	not applicable	dead
345	61	60-69	< median age	dead
346	59	50-59	< median age	dead
347	70	70-79	> median age	dead
348	56	50-59	< median age	dead
349	70	70-79	> median age	dead
350	57	50-59	< median age	dead
351	71	70-79	> median age	dead
352	78	70-79	> median age	living
353	93	90-99	> median age	living
354	70	70-79	> median age	dead
355	68	60-69	> median age	dead
356	54	50-59	< median age	dead
357	53	50-59	< median age	dead
358	79	70-79	> median age	dead
359	64	60-69	not applicable	dead
360	67	60-69	> median age	dead
361	69	60-69	not applicable	living
362	62	60-69	< median age	living
363	39	30-39	< median age	living
364	60	60-69	< median age	dead
365	72	70-79	> median age	living
366	57	50-59	< median age	dead
367	66	60-69	> median age	dead
368	61	60-69	< median age	dead
369	67	60-69	not applicable	dead
370	83	80-89	> median age	dead

S1 Table. Minimal data set.sav

	Survival_months	Survival_years	Predominant_staining_intensity
334	14,70	1,22	moderate staining
335	26,20	2,18	moderate staining
336	29,29	2,44	weak staining
337	10,68	,89	strong staining
338	3,95	,33	weak staining
339	84,69	7,06	weak staining
340	83,41	6,95	moderate staining
341	82,62	6,88	weak staining
342	10,65	,89	moderate staining
343	24,03	2,00	weak staining
344	,56	,05	strong staining
345	44,15	3,68	strong staining
346	1,71	,14	weak staining
347	27,78	2,32	weak staining
348	94,78	7,90	weak staining
349	11,57	,96	strong staining
350	47,31	3,94	weak staining
351	75,81	6,32	moderate staining
352	18,05	1,50	weak staining
353	78,05	6,50	moderate staining
354	21,34	1,78	moderate staining
355	7,00	,58	weak staining
356	78,90	6,58	weak staining
357	53,36	4,45	weak staining
358	43,50	3,62	strong staining
359	13,51	1,13	moderate staining
360	32,55	2,71	moderate staining
361	65,33	5,44	moderate staining
362	71,77	5,98	moderate staining
363	15,06	1,25	weak staining
364	29,06	2,42	moderate staining
365	,56	,05	weak staining
366	59,11	4,93	strong staining
367	29,59	2,47	moderate staining
368	13,74	1,15	weak staining
369	12,95	1,08	moderate staining
370	70,16	5,85	weak staining

S1 Table. Minimal data set.sav

	Percentage_of_positive_stained_cells	Immunoreactive_score
334	>80%	8
335	>80%	8
336	<10%	1
337	>80%	12
338	>80%	4
339	51-80%	3
340	51-80%	6
341	51-80%	3
342	>80%	8
343	10-50%	2
344	>80%	12
345	>80%	12
346	>80%	4
347	>80%	4
348	10-50%	2
349	>80%	12
350	>80%	4
351	>80%	8
352	51-80%	3
353	>80%	8
354	51-80%	6
355	>80%	4
356	51-80%	3
357	10-50%	2
358	>80%	12
359	>80%	8
360	>80%	8
361	>80%	8
362	51-80%	6
363	51-80%	3
364	>80%	8
365	>80%	4
366	>80%	12
367	>80%	8
368	>80%	4
369	>80%	8
370	51-80%	3

	Positive_IRS	Cutoff_IRS	Grouped_IRS
334	IRS >2	IRS ≤8	IRS 3-8
335	IRS >2	IRS ≤8	IRS 3-8
336	IRS ≤2	IRS ≤8	IRS 0-2
337	IRS >2	IRS >8	IRS 9-12
338	IRS >2	IRS ≤8	IRS 3-8
339	IRS >2	IRS ≤8	IRS 3-8
340	IRS >2	IRS ≤8	IRS 3-8
341	IRS >2	IRS ≤8	IRS 3-8
342	IRS >2	IRS ≤8	IRS 3-8
343	IRS ≤2	IRS ≤8	IRS 0-2
344	IRS >2	IRS >8	IRS 9-12
345	IRS >2	IRS >8	IRS 9-12
346	IRS >2	IRS ≤8	IRS 3-8
347	IRS >2	IRS ≤8	IRS 3-8
348	IRS ≤2	IRS ≤8	IRS 0-2
349	IRS >2	IRS >8	IRS 9-12
350	IRS >2	IRS ≤8	IRS 3-8
351	IRS >2	IRS ≤8	IRS 3-8
352	IRS >2	IRS ≤8	IRS 3-8
353	IRS >2	IRS ≤8	IRS 3-8
354	IRS >2	IRS ≤8	IRS 3-8
355	IRS >2	IRS ≤8	IRS 3-8
356	IRS >2	IRS ≤8	IRS 3-8
357	IRS ≤2	IRS ≤8	IRS 0-2
358	IRS >2	IRS >8	IRS 9-12
359	IRS >2	IRS ≤8	IRS 3-8
360	IRS >2	IRS ≤8	IRS 3-8
361	IRS >2	IRS ≤8	IRS 3-8
362	IRS >2	IRS ≤8	IRS 3-8
363	IRS >2	IRS ≤8	IRS 3-8
364	IRS >2	IRS ≤8	IRS 3-8
365	IRS >2	IRS ≤8	IRS 3-8
366	IRS >2	IRS >8	IRS 9-12
367	IRS >2	IRS ≤8	IRS 3-8
368	IRS >2	IRS ≤8	IRS 3-8
369	IRS >2	IRS ≤8	IRS 3-8
370	IRS >2	IRS ≤8	IRS 3-8

S1 Table. Minimal data set.sav

	Presence_of_strong_SI	Percentage_of_strong_SI
334	areas of strong staining intensity	10-50%
335	areas of strong staining intensity	<10%
336	no areas of strong staining intensity	no strong staining intensity
337	areas of strong staining intensity	10-50%
338	no areas of strong staining intensity	no strong staining intensity
339	no areas of strong staining intensity	no strong staining intensity
340	areas of strong staining intensity	<10%
341	no areas of strong staining intensity	no strong staining intensity
342	areas of strong staining intensity	10-50%
343	no areas of strong staining intensity	no strong staining intensity
344	areas of strong staining intensity	51-80%
345	areas of strong staining intensity	51-80%
346	no areas of strong staining intensity	no strong staining intensity
347	no areas of strong staining intensity	no strong staining intensity
348	no areas of strong staining intensity	no strong staining intensity
349	areas of strong staining intensity	51-80%
350	areas of strong staining intensity	<10%
351	areas of strong staining intensity	<10%
352	no areas of strong staining intensity	no strong staining intensity
353	areas of strong staining intensity	<10%
354	areas of strong staining intensity	<10%
355	no areas of strong staining intensity	no strong staining intensity
356	no areas of strong staining intensity	no strong staining intensity
357	no areas of strong staining intensity	no strong staining intensity
358	areas of strong staining intensity	51-80%
359	areas of strong staining intensity	<10%
360	areas of strong staining intensity	<10%
361	areas of strong staining intensity	10-50%
362	areas of strong staining intensity	<10%
363	areas of strong staining intensity	<10%
364	areas of strong staining intensity	10-50%
365	no areas of strong staining intensity	no strong staining intensity
366	areas of strong staining intensity	51-80%
367	areas of strong staining intensity	10-50%
368	no areas of strong staining intensity	no strong staining intensity
369	no areas of strong staining intensity	no strong staining intensity
370	no areas of strong staining intensity	no strong staining intensity

S1 Table. Minimal data set.sav

	Cohort	Histology	Grading
371	Ovarian carcinoma	serous high-grade	G3
372	Ovarian carcinoma	clear cell	G3
373	Ovarian carcinoma	serous high-grade	G3
374	Ovarian carcinoma	serous high-grade	G3
375	Ovarian carcinoma	serous high-grade	G3
376	Ovarian carcinoma	serous high-grade	G3
377	Ovarian carcinoma	serous high-grade	G3
378	Ovarian carcinoma	serous high-grade	G3
379	Ovarian carcinoma	serous high-grade	G3
380	Ovarian carcinoma	serous high-grade	G3
381	Ovarian carcinoma	serous high-grade	G3
382	Ovarian carcinoma	serous high-grade	G3
383	Ovarian carcinoma	serous high-grade	G3
384	Ovarian carcinoma	serous high-grade	G3
385	Ovarian carcinoma	serous high-grade	G3
386	Ovarian carcinoma	seromucinous	G3
387	Ovarian carcinoma	serous high-grade	G3
388	Ovarian carcinoma	serous high-grade	G3
389	Ovarian carcinoma	serous high-grade	G3
390	Ovarian carcinoma	serous high-grade	G3
391	Ovarian carcinoma	clear cell	G3
392	Ovarian carcinoma	undifferentiated	G3
393	Borderline ovarian tumor	serous	not applicable
394	Borderline ovarian tumor	serous	not applicable
395	Borderline ovarian tumor	serous	not applicable
396	Borderline ovarian tumor	serous	not applicable
397	Borderline ovarian tumor	serous	not applicable
398	Borderline ovarian tumor	mucinous	not applicable
399	Borderline ovarian tumor	mucinous	not applicable
400	Borderline ovarian tumor	mucinous	not applicable
401	Borderline ovarian tumor	serous	not applicable
402	Borderline ovarian tumor	serous	not applicable
403	Borderline ovarian tumor	serous	not applicable
404	Borderline ovarian tumor	serous	not applicable
405	Borderline ovarian tumor	serous	not applicable
406	Borderline ovarian tumor	serous	not applicable
407	Borderline ovarian tumor	mucinous	not applicable

S1 Table. Minimal data set.sav

	FIGO	Primary_tumor_expansion	Nodal_status	Distant_metastasis
371	FIGO III	T3	N0	MX/missing
372	FIGO IV	T3	N0	M1
373	FIGO III	T3	NX/missing	MX/missing
374	FIGO III	T3	N1	MX/missing
375	FIGO III	T3	N1	MX/missing
376	FIGO III	T3	N1	MX/missing
377	FIGO III	T3	N0	MX/missing
378	FIGO III	T3	N0	MX/missing
379	FIGO III	T3	N1	MX/missing
380	FIGO III	T3	N0	MX/missing
381	FIGO IV	T3	NX/missing	M1
382	FIGO IV	T3	N1	M1
383	FIGO III	T3	NX/missing	MX/missing
384	FIGO III	T3	N1	MX/missing
385	FIGO IV	T3	N1	M1
386	FIGO IV	T1	NX/missing	M1
387	FIGO IV	T3	N1	M1
388	FIGO III	T1	N1	MX/missing
389	FIGO III	T3	N0	MX/missing
390	FIGO III	T3	NX/missing	MX/missing
391	FIGO IV	T3	NX/missing	M1
392	FIGO III	T3	NX/missing	MX/missing
393	not applicable	not applicable	not applicable	not applicable
394	not applicable	not applicable	not applicable	not applicable
395	not applicable	not applicable	not applicable	not applicable
396	not applicable	not applicable	not applicable	not applicable
397	not applicable	not applicable	not applicable	not applicable
398	not applicable	not applicable	not applicable	not applicable
399	not applicable	not applicable	not applicable	not applicable
400	not applicable	not applicable	not applicable	not applicable
401	not applicable	not applicable	not applicable	not applicable
402	not applicable	not applicable	not applicable	not applicable
403	not applicable	not applicable	not applicable	not applicable
404	not applicable	not applicable	not applicable	not applicable
405	not applicable	not applicable	not applicable	not applicable
406	not applicable	not applicable	not applicable	not applicable
407	not applicable	not applicable	not applicable	not applicable

S1 Table. Minimal data set.sav

	Age	Grouped_age	Median_age_HGSC	Death
371	73	70-79	> median age	dead
372	38	30-39	not applicable	dead
373	68	60-69	> median age	living
374	61	60-69	< median age	living
375	79	70-79	> median age	dead
376	69	60-69	> median age	living
377	67	60-69	> median age	dead
378	60	60-69	< median age	living
379	44	40-49	< median age	dead
380	39	30-39	< median age	living
381	55	50-59	< median age	living
382	72	70-79	> median age	living
383	70	70-79	> median age	dead
384	64	60-69	> median age	dead
385	40	40-49	< median age	dead
386	63	60-69	not applicable	dead
387	67	60-69	> median age	dead
388	63	60-69	not applicable	living
389	67	60-69	> median age	dead
390	77	70-79	> median age	dead
391	42	40-49	not applicable	dead
392	66	60-69	not applicable	living
393	47	40-49	not applicable	not applicable
394	71	70-79	not applicable	not applicable
395	52	50-59	not applicable	not applicable
396	51	50-59	not applicable	not applicable
397	50	50-59	not applicable	not applicable
398	66	60-69	not applicable	not applicable
399	79	70-79	not applicable	not applicable
400	43	40-49	not applicable	not applicable
401	21	20-29	not applicable	not applicable
402	41	40-49	not applicable	not applicable
403	75	70-79	not applicable	not applicable
404	64	60-69	not applicable	not applicable
405	75	70-79	not applicable	not applicable
406	37	30-39	not applicable	not applicable
407	63	60-69	not applicable	not applicable

S1 Table. Minimal data set.sav

	Survival_months	Survival_years	Predominant_staining_intensity
371	1,28	,11	strong staining
372	86,93	7,24	moderate staining
373	27,78	2,32	moderate staining
374	75,55	6,30	moderate staining
375	5,13	,43	weak staining
376	13,08	1,09	weak staining
377	85,02	7,08	strong staining
378	83,87	6,99	weak staining
379	35,90	2,99	moderate staining
380	82,78	6,90	weak staining
381	12,33	1,03	weak staining
382	10,03	,84	weak staining
383	14,40	1,20	weak staining
384	77,65	6,47	moderate staining
385	18,61	1,55	weak staining
386	17,65	1,47	weak staining
387	30,48	2,54	weak staining
388	9,17	,76	weak staining
389	61,64	5,14	strong staining
390	16,27	1,36	no staining
391	26,99	2,25	strong staining
392	3,98	,33	weak staining
393	not applicable	not applicable	weak staining
394	not applicable	not applicable	moderate staining
395	not applicable	not applicable	moderate staining
396	not applicable	not applicable	moderate staining
397	not applicable	not applicable	weak staining
398	not applicable	not applicable	moderate staining
399	not applicable	not applicable	moderate staining
400	not applicable	not applicable	weak staining
401	not applicable	not applicable	weak staining
402	not applicable	not applicable	weak staining
403	not applicable	not applicable	moderate staining
404	not applicable	not applicable	moderate staining
405	not applicable	not applicable	moderate staining
406	not applicable	not applicable	weak staining
407	not applicable	not applicable	moderate staining

S1 Table. Minimal data set.sav

	Percentage_of_positive_stained_cells	Immunoreactive_score
371	>80%	12
372	>80%	8
373	>80%	8
374	51-80%	6
375	10-50%	2
376	>80%	4
377	>80%	12
378	<10%	1
379	>80%	8
380	51-80%	3
381	>80%	4
382	10-50%	2
383	>80%	4
384	51-80%	6
385	10-50%	2
386	<10%	1
387	>80%	4
388	>80%	4
389	>80%	12
390	no staining	0
391	>80%	12
392	10-50%	2
393	51-80%	3
394	<10%	2
395	10-50%	4
396	10-50%	4
397	51-80%	3
398	51-80%	6
399	>80%	8
400	<10%	1
401	<10%	1
402	51-80%	3
403	10-50%	4
404	10-50%	4
405	10-50%	4
406	10-50%	2
407	51-80%	6

S1 Table. Minimal data set.sav

	Positive_IRS	Cutoff_IRS	Grouped_IRS
371	IRS >2	IRS >8	IRS 9-12
372	IRS >2	IRS ≤8	IRS 3-8
373	IRS >2	IRS ≤8	IRS 3-8
374	IRS >2	IRS ≤8	IRS 3-8
375	IRS ≤2	IRS ≤8	IRS 0-2
376	IRS >2	IRS ≤8	IRS 3-8
377	IRS >2	IRS >8	IRS 9-12
378	IRS ≤2	IRS ≤8	IRS 0-2
379	IRS >2	IRS ≤8	IRS 3-8
380	IRS >2	IRS ≤8	IRS 3-8
381	IRS >2	IRS ≤8	IRS 3-8
382	IRS ≤2	IRS ≤8	IRS 0-2
383	IRS >2	IRS ≤8	IRS 3-8
384	IRS >2	IRS ≤8	IRS 3-8
385	IRS ≤2	IRS ≤8	IRS 0-2
386	IRS ≤2	IRS ≤8	IRS 0-2
387	IRS >2	IRS ≤8	IRS 3-8
388	IRS >2	IRS ≤8	IRS 3-8
389	IRS >2	IRS >8	IRS 9-12
390	IRS ≤2	IRS ≤8	IRS 0-2
391	IRS >2	IRS >8	IRS 9-12
392	IRS ≤2	IRS ≤8	IRS 0-2
393	IRS >2	IRS ≤8	IRS 3-8
394	IRS ≤2	IRS ≤8	IRS 0-2
395	IRS >2	IRS ≤8	IRS 3-8
396	IRS >2	IRS ≤8	IRS 3-8
397	IRS >2	IRS ≤8	IRS 3-8
398	IRS >2	IRS ≤8	IRS 3-8
399	IRS >2	IRS ≤8	IRS 3-8
400	IRS ≤2	IRS ≤8	IRS 0-2
401	IRS ≤2	IRS ≤8	IRS 0-2
402	IRS >2	IRS ≤8	IRS 3-8
403	IRS >2	IRS ≤8	IRS 3-8
404	IRS >2	IRS ≤8	IRS 3-8
405	IRS >2	IRS ≤8	IRS 3-8
406	IRS ≤2	IRS ≤8	IRS 0-2
407	IRS >2	IRS ≤8	IRS 3-8

S1 Table. Minimal data set.sav

	Presence_of_strong_SI	Percentage_of_strong_SI
371	areas of strong staining intensity	51-80%
372	areas of strong staining intensity	<10%
373	no areas of strong staining intensity	no strong staining intensity
374	areas of strong staining intensity	<10%
375	no areas of strong staining intensity	no strong staining intensity
376	areas of strong staining intensity	<10%
377	areas of strong staining intensity	>80%
378	no areas of strong staining intensity	no strong staining intensity
379	areas of strong staining intensity	<10%
380	no areas of strong staining intensity	no strong staining intensity
381	no areas of strong staining intensity	no strong staining intensity
382	no areas of strong staining intensity	no strong staining intensity
383	no areas of strong staining intensity	no strong staining intensity
384	areas of strong staining intensity	<10%
385	no areas of strong staining intensity	no strong staining intensity
386	no areas of strong staining intensity	no strong staining intensity
387	no areas of strong staining intensity	no strong staining intensity
388	no areas of strong staining intensity	no strong staining intensity
389	areas of strong staining intensity	>80%
390	no areas of strong staining intensity	no strong staining intensity
391	areas of strong staining intensity	10-50%
392	no areas of strong staining intensity	no strong staining intensity
393	areas of strong staining intensity	<10%
394	areas of strong staining intensity	<10%
395	areas of strong staining intensity	<10%
396	areas of strong staining intensity	<10%
397	areas of strong staining intensity	<10%
398	areas of strong staining intensity	<10%
399	areas of strong staining intensity	10-50%
400	no areas of strong staining intensity	no strong staining intensity
401	areas of strong staining intensity	<10%
402	areas of strong staining intensity	10-50%
403	areas of strong staining intensity	<10%
404	areas of strong staining intensity	<10%
405	areas of strong staining intensity	<10%
406	no areas of strong staining intensity	no strong staining intensity
407	areas of strong staining intensity	10-50%

S1 Table. Minimal data set.sav

	Cohort	Histology	Grading
408	Borderline ovarian tumor	seromucinous	not applicable
409	Borderline ovarian tumor	serous	not applicable
410	Borderline ovarian tumor	serous	not applicable
411	Borderline ovarian tumor	serous	not applicable
412	Control group	not applicable	not applicable
413	Control group	not applicable	not applicable
414	Control group	not applicable	not applicable
415	Control group	not applicable	not applicable
416	Control group	not applicable	not applicable
417	Control group	not applicable	not applicable
418	Control group	not applicable	not applicable
419	Control group	not applicable	not applicable
420	Control group	not applicable	not applicable
421	Control group	not applicable	not applicable
422	Control group	not applicable	not applicable
423	Control group	not applicable	not applicable
424	Control group	not applicable	not applicable
425	Control group	not applicable	not applicable
426	Control group	not applicable	not applicable
427	Control group	not applicable	not applicable
428	Control group	not applicable	not applicable
429	Control group	not applicable	not applicable
430	Control group	not applicable	not applicable

S1 Table. Minimal data set.sav

	FIGO	Primary_tumor_expansion	Nodal_status	Distant_metastasis
408	not applicable	not applicable	not applicable	not applicable
409	not applicable	not applicable	not applicable	not applicable
410	not applicable	not applicable	not applicable	not applicable
411	not applicable	not applicable	not applicable	not applicable
412	not applicable	not applicable	not applicable	not applicable
413	not applicable	not applicable	not applicable	not applicable
414	not applicable	not applicable	not applicable	not applicable
415	not applicable	not applicable	not applicable	not applicable
416	not applicable	not applicable	not applicable	not applicable
417	not applicable	not applicable	not applicable	not applicable
418	not applicable	not applicable	not applicable	not applicable
419	not applicable	not applicable	not applicable	not applicable
420	not applicable	not applicable	not applicable	not applicable
421	not applicable	not applicable	not applicable	not applicable
422	not applicable	not applicable	not applicable	not applicable
423	not applicable	not applicable	not applicable	not applicable
424	not applicable	not applicable	not applicable	not applicable
425	not applicable	not applicable	not applicable	not applicable
426	not applicable	not applicable	not applicable	not applicable
427	not applicable	not applicable	not applicable	not applicable
428	not applicable	not applicable	not applicable	not applicable
429	not applicable	not applicable	not applicable	not applicable
430	not applicable	not applicable	not applicable	not applicable

S1 Table. Minimal data set.sav

	Age	Grouped_age	Median_age_HGSC	Death
408	48	40-49	not applicable	not applicable
409	48	40-49	not applicable	not applicable
410	67	60-69	not applicable	not applicable
411	61	60-69	not applicable	not applicable
412	59	50-59	not applicable	not applicable
413	59	50-59	not applicable	not applicable
414	51	50-59	not applicable	not applicable
415	51	50-59	not applicable	not applicable
416	54	50-59	not applicable	not applicable
417	76	70-79	not applicable	not applicable
418	76	70-79	not applicable	not applicable
419	69	60-69	not applicable	not applicable
420	69	60-69	not applicable	not applicable
421	79	70-79	not applicable	not applicable
422	56	50-59	not applicable	not applicable
423	56	50-59	not applicable	not applicable
424	47	40-49	not applicable	not applicable
425	74	70-79	not applicable	not applicable
426	74	70-79	not applicable	not applicable
427	53	50-59	not applicable	not applicable
428	53	50-59	not applicable	not applicable
429	63	60-69	not applicable	not applicable
430	63	60-69	not applicable	not applicable

S1 Table. Minimal data set.sav

	Survival_months	Survival_years	Predominant_staining_intensity
408	not applicable	not applicable	moderate staining
409	not applicable	not applicable	weak staining
410	not applicable	not applicable	moderate staining
411	not applicable	not applicable	weak staining
412	not applicable	not applicable	weak staining
413	not applicable	not applicable	weak staining
414	not applicable	not applicable	weak staining
415	not applicable	not applicable	weak staining
416	not applicable	not applicable	moderate staining
417	not applicable	not applicable	weak staining
418	not applicable	not applicable	weak staining
419	not applicable	not applicable	weak staining
420	not applicable	not applicable	weak staining
421	not applicable	not applicable	weak staining
422	not applicable	not applicable	weak staining
423	not applicable	not applicable	weak staining
424	not applicable	not applicable	weak staining
425	not applicable	not applicable	moderate staining
426	not applicable	not applicable	weak staining
427	not applicable	not applicable	weak staining
428	not applicable	not applicable	weak staining
429	not applicable	not applicable	weak staining
430	not applicable	not applicable	moderate staining

S1 Table. Minimal data set.sav

	Percentage_of_positive_stained_cells	Immunoreactive_score
408	51-80%	6
409	51-80%	3
410	51-80%	6
411	51-80%	3
412	51-80%	3
413	10-50%	2
414	10-50%	2
415	10-50%	2
416	<10%	2
417	10-50%	2
418	10-50%	2
419	10-50%	2
420	<10%	1
421	10-50%	2
422	10-50%	2
423	51-80%	3
424	10-50%	2
425	51-80%	6
426	10-50%	2
427	10-50%	2
428	<10%	1
429	51-80%	3
430	51-80%	6

	Positive_IRS	Cutoff_IRS	Grouped_IRS
408	IRS >2	IRS ≤8	IRS 3-8
409	IRS >2	IRS ≤8	IRS 3-8
410	IRS >2	IRS ≤8	IRS 3-8
411	IRS >2	IRS ≤8	IRS 3-8
412	IRS >2	IRS ≤8	IRS 3-8
413	IRS ≤2	IRS ≤8	IRS 0-2
414	IRS ≤2	IRS ≤8	IRS 0-2
415	IRS ≤2	IRS ≤8	IRS 0-2
416	IRS ≤2	IRS ≤8	IRS 0-2
417	IRS ≤2	IRS ≤8	IRS 0-2
418	IRS ≤2	IRS ≤8	IRS 0-2
419	IRS ≤2	IRS ≤8	IRS 0-2
420	IRS ≤2	IRS ≤8	IRS 0-2
421	IRS ≤2	IRS ≤8	IRS 0-2
422	IRS ≤2	IRS ≤8	IRS 0-2
423	IRS >2	IRS ≤8	IRS 3-8
424	IRS ≤2	IRS ≤8	IRS 0-2
425	IRS >2	IRS ≤8	IRS 3-8
426	IRS ≤2	IRS ≤8	IRS 0-2
427	IRS ≤2	IRS ≤8	IRS 0-2
428	IRS ≤2	IRS ≤8	IRS 0-2
429	IRS >2	IRS ≤8	IRS 3-8
430	IRS >2	IRS ≤8	IRS 3-8

S1 Table. Minimal data set.sav

	Presence_of_strong_SI	Percentage_of_strong_SI
408	areas of strong staining intensity	10-50%
409	areas of strong staining intensity	<10%
410	areas of strong staining intensity	10-50%
411	areas of strong staining intensity	<10%
412	no areas of strong staining intensity	no strong staining intensity
413	no areas of strong staining intensity	no strong staining intensity
414	no areas of strong staining intensity	no strong staining intensity
415	no areas of strong staining intensity	no strong staining intensity
416	areas of strong staining intensity	<10%
417	no areas of strong staining intensity	no strong staining intensity
418	areas of strong staining intensity	<10%
419	no areas of strong staining intensity	no strong staining intensity
420	no areas of strong staining intensity	no strong staining intensity
421	no areas of strong staining intensity	no strong staining intensity
422	areas of strong staining intensity	<10%
423	no areas of strong staining intensity	no strong staining intensity
424	no areas of strong staining intensity	no strong staining intensity
425	areas of strong staining intensity	<10%
426	no areas of strong staining intensity	no strong staining intensity
427	areas of strong staining intensity	<10%
428	areas of strong staining intensity	<10%
429	no areas of strong staining intensity	no strong staining intensity
430	areas of strong staining intensity	<10%
